# Decade-long monitoring of seismic velocity changes at the Irpinia fault system (southern Italy) reveals pore pressure pulsations

**DOI:** 10.1038/s41598-022-05365-x

**Published:** 2022-01-24

**Authors:** G. De Landro, O. Amoroso, G. Russo, N. D’Agostino, R. Esposito, A. Emolo, A. Zollo

**Affiliations:** 1 Department of Physics “E. Pancini”, University of Naples ‘Federico II’, Naples, Italy; 2 Department of Physics “E.R. Caianiello”, University of Salerno, Fisciano (SA), Italy; 3 National Institute of Geophysics and Volcanology, Rome, Italy; 4 Formerly Department of Physics “E. Pancini”, University of Naples ‘Federico II’, Naples, Italy

**Keywords:** Seismology, Solid Earth sciences, Geophysics, Natural hazards

## Abstract

Several studies have monitored crustal seismic velocity changes and attempted to relate them to the stress state and physical properties in volume embedding fault systems. The aim is to provide constraints on fault system dynamics and earthquake triggering mechanisms. Here, we reconstruct the spatiotemporal (4D) seismic velocity images of volume embedding the Irpinia fault system (IFS, South Italy), which originated the 1980 M_s_ 6.9 multi-segmented ruptures. By inverting data from more than ten years of continuous seismicity monitoring, we retrieved time-constant velocity anomalies, whose shapes correlate well with crustal lithology, while time-changing (up to 20%) velocity anomalies are mapped in the central region. Here, the Vp-to-Vs changes at depths of 1–5 km and 8–12 km correlate well with groundwater recharge and geodetic displacement during the same time interval. This correlation provides evidence for the existence of pulsating, pore pressure changes induced by groundwater recharge processes in a deep volume (8–12 km of depth), fractured and saturated with a predominant gas phase (likely CO_2_). We suggest that tomographic measurements of the Vp-to-Vs spatiotemporal changes are a suitable proxy to track the pore pressure evolution at depth in highly sensitive regions of fault systems.

## Introduction

The contribution of pore fluid pressure changes to triggering earthquakes at different scales of rupture is recognized worldwide^1^ and has been invoked in several Italian Apennine cases^2–8^. In this area, in the past decade, several works investigated the involvement of fluids in the preparatory phase of moderate to large earthquakes by monitoring the changes in medium parameters^3,5,6^, hydrogeochemical properties of gas emission^7^ and/or water-table and spring discharge^8,9^. The results of these studies, while looking at different parameters, enforced the connection between crustal fluids dynamic and earthquake triggering by invoking the fluid overpressure (pore pressure above the hydrostatic) as primary mechanism^3–5,9^ and suggested the needing of a multi-parametric analysis to investigate these complex processes.

**Figure 1:**
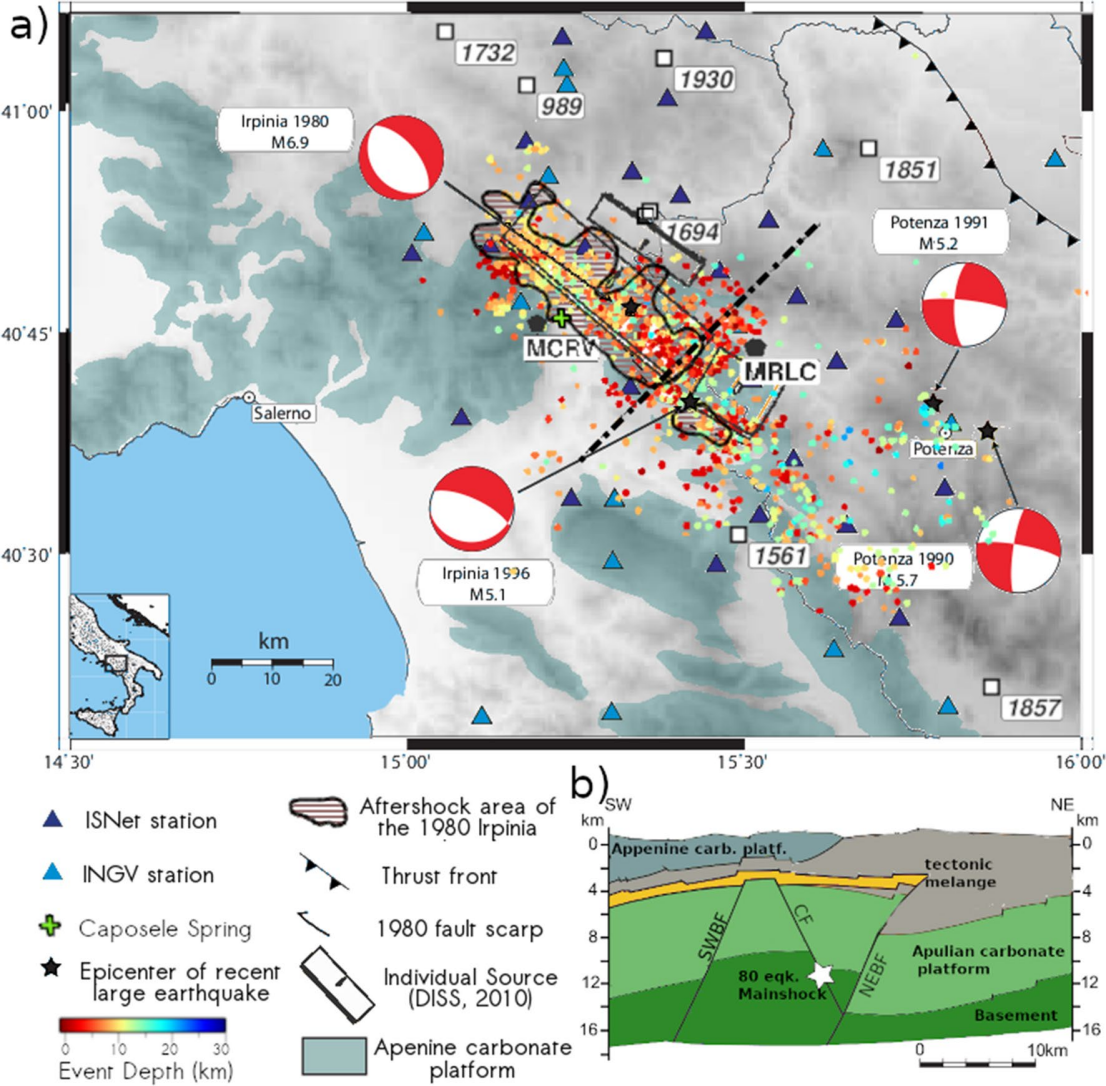
Tectonic setting of the Irpinia Fault System. (**a**) Geological sketch map. The white squares indicate the locations of the main historical and instrumental earthquakes; for the most recent earthquakes, the focal mechanisms are reported. The black boxes are the Irpinia Faults as reported by the Database of Individual Seismogenic Sources^11^. The green contoured areas represent the Apennine carbonate rocks. The contoured black area represents the aftershock area of the 1980 Irpinia earthquake. The dashed line is the SW–NE section shown in panel (**b**). The circles indicate the events used in this study that are coloured according to their depth. The map was generated with the Generic Mapping Tool Software (GMT ver.4; https://www.soest.hawaii.edu/gmt/). (**b**) Geological cross-section along the profile reported in the map in (**a**); *SWBF* SW boundary fault, *CF* central fault, *NEBF* NE boundary fault.

The Irpinia fault system (IFS), which is located in the southern part of the Apennine chain (Italy; Fig. 1a), generated the largest Italian event in the last 100 years: the 1980 M_s_ 6.9 Irpinia earthquake. The earthquake involved a complex three-segment fault structure aligned along the Apennines chain, two of which were activated at 0 s and 20 s and dip NE, and the third that was activated 40 s after the first one and dips SW^10^.

Since 2005, the IFS has been monitored by the Irpinia Seismic Network (ISNet; blue triangles in Fig. 1a), which is composed of 33 stations and deployed for early warning purposes^12^. The catalogue of micro-seismicity shows a stable low magnitude (0.5 < M_L_ < 3.5) pattern, which is spatially located along the whole system, is widespread inside the volume between the boundary faults (i.e., southwest and northeast boundary faults in Fig. 1b, referred to as SWBF and NEBF, respectively^13^), and allows an investigation of the interaction processes between crustal fluids and micro-seismicity at the IFS with different approaches^14^.

Amoroso et al.^15^ retrieved 3D tomographic P and S velocity images showing that the hypocentral volume of the 1980 Irpinia earthquake was characterized by a high Vp-to-Vs ratio, which was interpreted as being linked to the presence of fluid-saturated rocks. Similar results were obtained by Improta et al.^2^. Amoroso et al.^16^, using the data of Zollo et al.^17^, coupled attenuated tomography images with rock physical modelling to confirm the presence of fluids in the whole volume where seismicity occurs and to provide an estimate of the fluid type and saturation in the hypocentral volume of the 1980 earthquake, thereby finally recognizing that fluids may generate high pore pressure. D'Agostino et al.^18^ showed that geodetic transient deformation and seismicity are modulated by phases of recharge of large karst aquifers hosted by Mesozoic carbonates. In particular, in the case of the IFS, the steady-state ~ 3 mm/year extension across the Apennines is enhanced (reduced) by high transient horizontal extension driven by a high (low) hydraulic head (inferred from the temporal pattern of spring discharge) in the karst. Picozzi et al.^19^, tracing the spatiotemporal evolution of earthquake parameters, identified two long-lasting phases in the seismicity pattern associated with transient pore pressure perturbations.

The established link between fluids and seismicity within the IFS underlines the importance of fluid tracking for a better characterization of medium rock physical properties, seismicity, and, possibly, hazard reduction. In this paper, we used a 10-year dataset of IFS micro-seismicity from 2005 to 2016 to track the spatiotemporal changes in elastic medium properties by 4D tomography (used for monitoring purposes in tectonic zones^3,6,20^; volcanic systems^21^; and exploitation areas^22^). To our knowledge, this work represents the first example of time-lapse tomography applied to a fault system with a high seismogenic potential for a decade-long interval.

To unravel the complex dynamics of the fault system and assess the role of hydrological forcing on the elastic properties of the crust, we tracked velocity changes between epochs and compared the temporal evolution of the Vp-to-Vs ratio in two volumes of interest, located in the central part of the IFS and between depths of 1–5 km and 8–12 km, with hydrological and geodetic observables.

We found a strong correlation between the velocity changes and hydraulic head, even for the deeper-volume, highly responsive to hydro head, where the 1980 Irpinia earthquake enucleated; thus, we interpreted it as fractured and saturated by over-pressurized fluids. We constructed a conceptual model to interpret the retrieved correlation that connects hydrological forcing, stress perturbations and velocity changes. The described mechanisms may represent an important part of the IFS dynamics that may play a key role in triggering intense current micro-seismic activity.

## 3D and 4D images of the volume embedding the IFS

We considered the ISNet catalogue from August 2005 to October 2016. It consists of 1948 low magnitude (0.5 < ML < 3.2) events. For these earthquakes, we used accurate pickings manually revised by the University of Naples Federico II (UNINA) and by the National Institute of Geophysics and Volcanology (INGV) that manage ISNET and the Italian seismic surveillance network, respectively. To obtain the initial event locations, we localized the events using the NLLoc code^23^ and the 1D velocity model retrieved for the IFS crustal volume by Matrullo et al.^24^. Then, we performed a selection based on the quality of locations that mainly depends on the stations/event layout and the noise level at the stations (Ref.^25^; see the Supplementary Information, SI). A total of 1425 events finally provided 12,598 P picks and 5834 S picks (see Supplementary Fig. S1 in the SI).

Using these data, we performed a first arrival time tomographic inversion using an iterative, linearized method^26^ and a multiscale approach (for further details on the inversion strategy, see the SI and Supplementary Figs. S2, S3 and S4).

**Figure 2:**
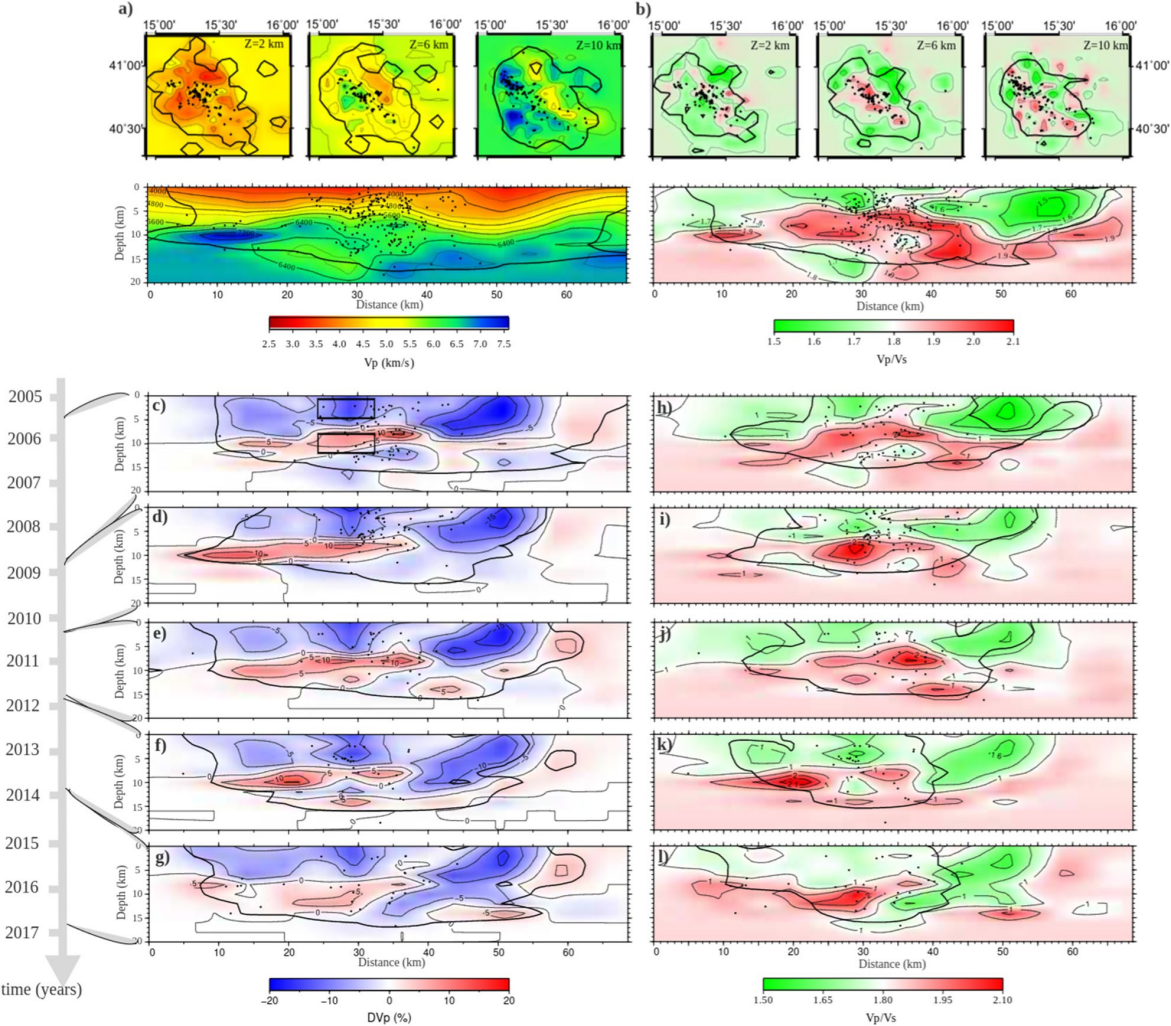
Three-dimensional tomographic models. (**a**) V_P_ and (**b**) Vp-to-Vs horizontal slices for parametrization 6 × 6 × 2 km^3^ at different depths (2, 6 and 10 km) and cross-sections located in Fig. 1a. The black curves delimit the well-resolved model regions according to the resolution analysis (Supplementary Figs. S3–S4). The black dots represent the earthquakes located in a range of 1 km around each layer depth. (**c**–**g**) Vp variations with respect to the 1D initial model and (**h**–**j**) Vp-to-Vs model cross-sections in the different epochs. The grey arrow shows the temporal axis and the temporal reference of each epoch. The black curves delimit the well-resolved model regions according to the resolution analysis (Supplementary Figs. S6–S7). The black squares in panel c represent the two volumes of interest. The maps were generated with the Generic Mapping Tool Software (GMT ver.4; https://www.soest.hawaii.edu/gmt/).

In Fig. 2, we show the 6 × 6 × 3 km^3^ Vp (panel a) and Vp-to-Vs (panel b) models (which we hereafter refer to as 3DWM, i.e., the entire models; Supplementary Fig. S5 in SI). The black contours mark the resolved area for each depth assessed by evaluating the resolution matrix elements and the ray density for each node of the grid (for further details on resolution assessment see the SI and Supplementary Fig. S6). These contours correspond to a value greater than or equal to 0.9 for the diagonal elements of the resolution matrix.

The section of P-wave velocity along a SW–NE direction (Fig. 2a), orthogonal to the Apennine chain, shows that the tomographic model delineates the main well-known lithological discontinuities (see the geological section in Fig. 1b; Ref.^15^). The section in Fig. 2b shows a diffuse high Vp-to-Vs ratio within the Apulian Platform carbonates and between the boundary faults (Fig. 1b; Ref.^15^).

The entire period was then divided into consecutive time windows (which we hereafter refer to as “epochs''). To optimize each epoch duration, we recursively incremented the event number and selected the event number allowing us to reach a good resolution in the volume of interest and, at the same time, to maximize the temporal sampling. The resolution in each epoch was assessed by evaluating the resolution matrix elements and the ray density around each node (Supplementary Figs. S7,S8). This procedure is feasible because the IFS background seismicity insists on the same structure over time (Supplementary Fig. S9), but since it is non-time-uniform, the epochs span different time periods (Table 1). At the end of this optimization analysis we don’t expect exactly the same resolved volumes in each epoch, but to be able to reach the same resolution in the volumes of interest. Indeed, the crucial aspect in our analysis is the comparison between velocity features that are inside the resolved area for all the epochs.

**Table 1:** Epochs subdivision (time format mm/yyyy). Table of the subdivision of the entire time period in 5 epochs of variable duration, based on resolution requirement of 4D tomographic images.

Epoch 1	Epoch 2	Epoch 3	Epoch 4	Epoch 5
8/2005–9/2008	9/2008–2/2010	2/2010–8/2011	8/2011–7/2013	7/2013–10/2016

For each epoch, tomographic inversion was performed using the multiscale approach (see the SI and Supplementary Figs. S10–S14). Moreover, we adopted an initial 1D model obtained by averaging the 3DWM at each depth. In Fig. 2d–k, we show the resulting models for the 6 × 6 × 2 km^3^ parametrization.

The 3D Vp variations with respect to the 1D initial model (Fig. 2c–g) and Vp-to-Vs (Fig. 2h–l) images for each epoch were compared and matched in the common best resolved volume (black contoured volume in Fig. 2c–l), with the aim of identifying possible seismic velocity changes over time.

At intermediate depths, some specific regions (IFS central region at a depth of 1–5 km and NE region at a depth of 1–8 km) are characterized by low Vp and low Vp-to-Vs (< 1.75) values that are also present in the entire model. These anomalies have been previously associated with the fractured carbonates of the Apennine Platform, to the SW and to Lagonegro Basin strata to the NE^2,7^. Among these regions, the seismic velocity of the NE does not show significant changes over time, which we interpret as the main unperturbed effect of lithology. On the other hand, in the central volume, the Vp-to-Vs ratio shows temporal changes up to 7% among the epochs.

In the deeper IFS central region, between 8 and 12 km of depth, we identify significant changes, up to 20% (from 1.75 to 2.10), in the Vp-to-Vs ratio over time. In particular, the larger variations in Vp-to-Vs values (see, for instance, the low Vp-to-Vs anomaly in the fourth epoch, panel k in Fig. 2) are located in the volume between the SW and NE Boundary Faults where the Irpinia 1980 earthquake enucleated (white star in Fig. 1b) and where Amoroso et al.^16^ retrieved a high P-wave quality factor. This parameter measures the relative fraction of energy dissipated for frictional mechanisms and is very highly responsive to fluid presence in the host medium.

In the following, we focus our analysis on the two volumes of interest in the IFS central region characterized by Vp-to-Vs changes (i.e., one “shallower” between 1 and 5 km of depth and the other “deeper” between 8 and 12 km of depth; black boxes in Fig. 2c).

## Vp-to-Vs changes, spring discharge and crustal deformation

**Figure 3:**
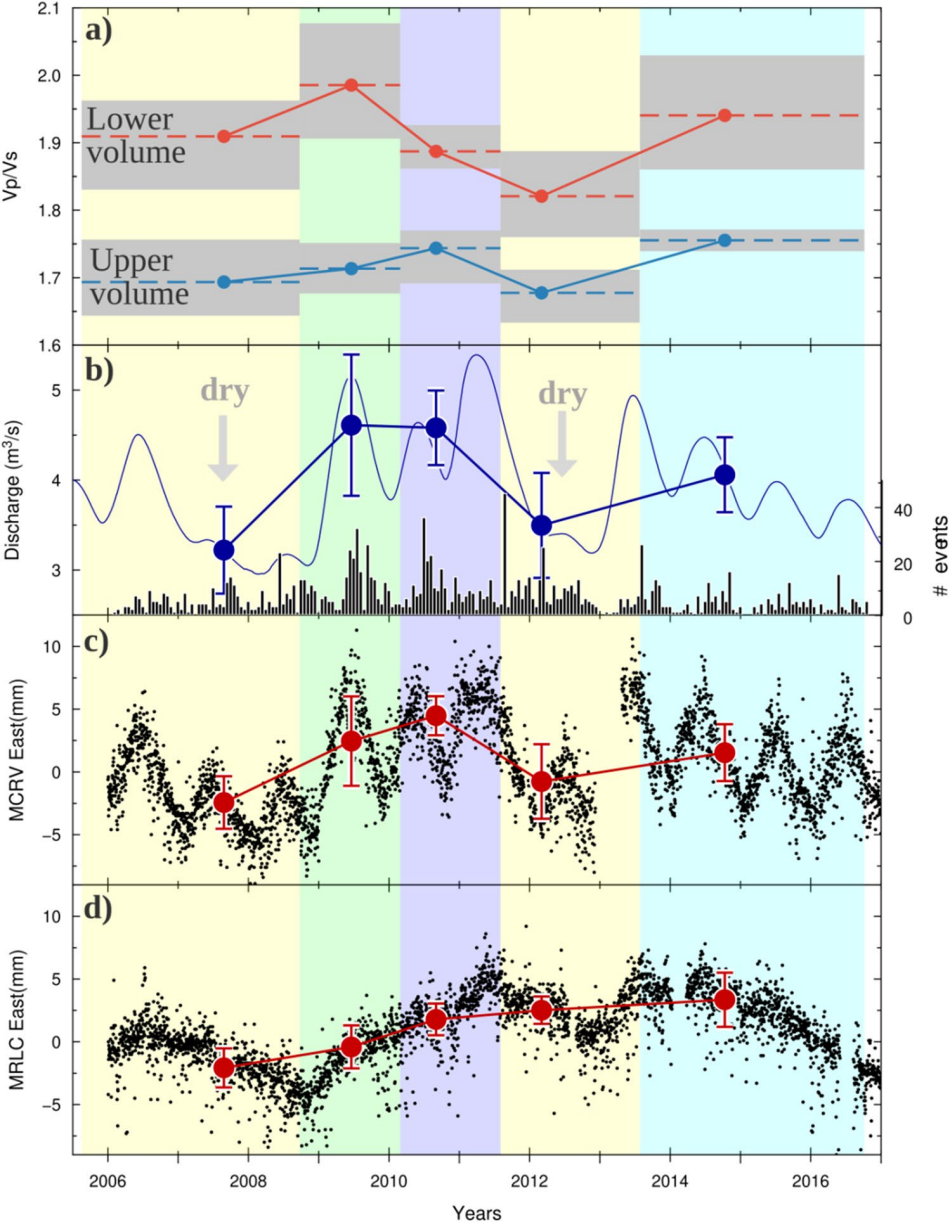
1D temporal evolution of Vp-to-Vs, discharge and deformation. (**a**) 1D temporal evolution of Vp-to-Vs ratios for each epoch in shallower (blue curve) and deeper (red curve) volumes. The ratio values are extrapolated at 20%, 50% and 80% of the velocity value cumulative function (Supplementary Figs. S9–S10). (b**)** Spring discharge of the Caposele spring (in blue, location in Fig. 1a). Arrows indicate drought years. The black histogram represents the temporal evolution of seismicity in each epoch. (**c**,**d**) Detrended eastern component time series of MCRV and MRLC GPS stations (from D’Agostino et al., 2018; red circles; locations in Fig. 1a). This figure was generated with the Generic Mapping Tool Software (GMT ver.4; https://www.soest.hawaii.edu/gmt/).

In the two volumes of interest, we extrapolated the Vp-to-Vs ratios for each epoch (at 20%, 50% and 80% of the cumulative distribution of velocity, Supplementary Figs. S15–S16) and plotted the 1D velocity changes versus time in Fig. 3a. In order to enforce the reliability of the reconstructed 1D velocity changes, we carried out a sensitivity test proving that no lack of resolution, due to epoch construction, affects the volumes of interest in 4D images (see SI and Supplementary Fig. S17).

In the shallower central volume, the Vp-to-Vs ratio (blue curve in Fig. 3a) ranges between 1.65 and 1.75 (variation up to 7% with respect to the smallest value). It slightly increases between the first three epochs, significantly decreases in the fourth epoch (from the mean value of 1.70 to 1.65) and then increases again in the last epoch. These variations are mainly due to Vs changes, especially around the minimum of the fourth epoch, while Vp does not show significant changes (less than 5%, Fig. 2d–h).

In the deeper central volume, the Vp-to-Vs temporal changes (red curve in Fig. 3a) are larger (up to 20%) than those in the shallower volume. The ratio ranges between 1.75 and 2.10, increasing from the first to the second epoch and from the fourth to the fifth epoch. From the second to the fourth epoch, the ratio decreases. In this case, the Vp-to-Vs changes are mainly related to Vp changes, up to 15–20% around the minimum of the fourth epoch (Fig. 2f).

To analyse the relation between the elastic property variations and the hydrological forcing operated by a variable hydraulic head in the karst aquifer, we compared the Vp-to-Vs curves with time series of (1) the spring discharge of the Caposele spring (Fig. 3b; see Fig. 1a for location), which is used as a proxy for hydraulic head in the karst reservoir, and (2) the detrended eastern component of the Global Positioning System (GPS) displacements from stations located at Calabritto (MCRV) and Muro Lucano (MRLC) (Fig. 3c,d; see Fig. 1a for their locations) for the same analysed period. To define comparable values of those observables for each epoch, we extracted the observable measures corresponding to the occurrence of seismicity (Fig. 3b, panel bottom), and we computed the median of their distribution (black dots in Fig. 3b–d).

The Caposele spring (417 m a.s.l.; Fig. 1a) has a mean annual discharge of approximately 4 m^3^/s, with a large seasonal variability (Fig. 3b; Ref.^27^): the maximum discharge occurs in spring–summer (May–July), whereas the minimum discharge takes place during the rainy months (November–December). During the analysed period, 2008 and 2012 were drought years, i.e., they were characterized by a reduced discharge during the entire hydrological year (arrows in Fig. 3b).

Time series of crustal displacements from permanent GPS stations show the occurrence of seasonal and multiannual modulation of extensional strain transients near karst aquifers with a strong temporal correlation with phases of groundwater recharge^18,28^. This correlation and the absence of a temporal shift between the two trends suggests that highly fractured, hydraulically connected shallow crustal materials (0–3 km) expand horizontally in a NE–SW direction by diffuse crack opening as a response to the increase in hydraulic head in karst aquifers^18^. It is interesting to note that not only are the Vp-to-Vs changes in the shallower volume (Fig. 3a, blue curve) correlated with the discharge/displacement trends but also are the changes in the deeper volume (Fig. 3a, red curve). Therefore, the variation in hydraulic head in surficial aquifers (2–3 km deep) and the consequent dynamic deformation affect the medium elastic properties at greater depths (up to 8–12 km) in a region highly sensitive to groundwater recharge processes. A time shift is not visible between the spring discharge curve and Vp-to-Vs ratio variations at depth. This indicates that the time lag of velocity changes is smaller than the tomographic epoch time interval, the smaller of which is 13 months. This evidence is consistent with D’Agostino et al.^18^, who observed a time lag of 0.1–0.2 years between spring discharges and seismicity at depths of 6–12 km.

## Interpretative model

**Figure 4:**
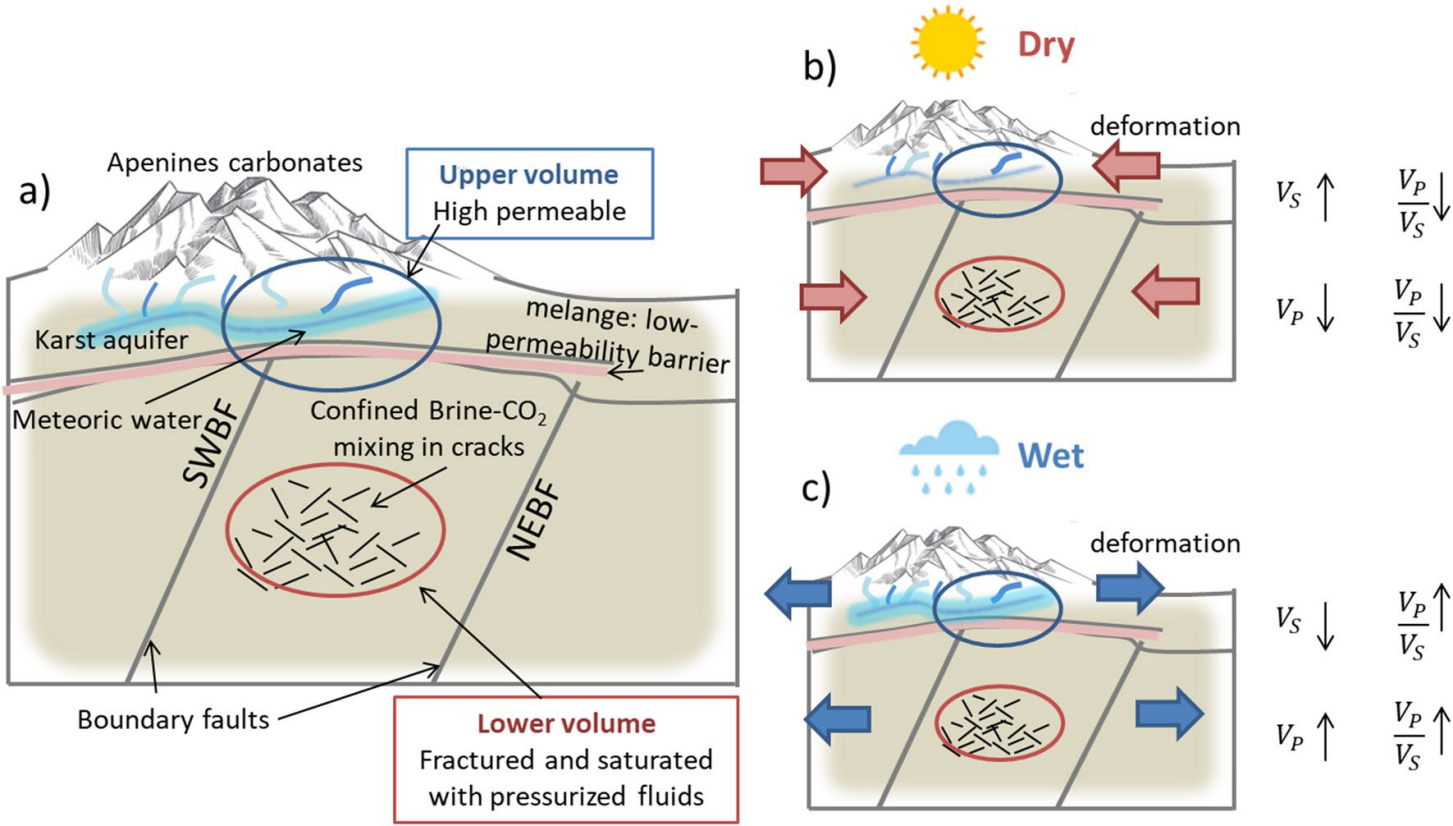
Interpretative conceptual model. (**a**) Schematic section of the conceptual model with the position of the shallower volume (blue circle) and deeper volume (red circle) and the main lithological features (i.e., Apennine carbonates, karst aquifer, mélange and boundary faults). (**b**) Dry and (**c**) wet conditions and the model behaviour in terms of hydraulic head, stress state (arrows) and velocity variations. The arrows represent the ground deformation consequent to the hydrological condition.

To explain the correlation between hydraulic forcing and seismic velocity variations in the crustal volume embedding the IFS, we propose an interpretative model that accounts for the rock volume rheology, pressure conditions, porosity, and fluid content type and saturation (Fig. 4a).

The SW–NE geological cross-section (Fig. 1b) shows the shallower volume (blue circle in Fig. 4a) between the Apennines carbonates (light green surfaces in Fig. 1a,b) and the tectonic mélange (yellow strata in Fig. 1b). Intensely fractured and permeable Mesozoic limestones host shallow karst aquifers^29^. Celico^30^ showed that the circulation of meteoric waters extends to a depth of at least ~ 3 km, which is consistent with a highly permeable, saturated shallow crust under hydrostatic pressure conditions.

The deeper volume (red circle in Fig. 4a) is located beneath the mélange and between the two boundary faults (SWBF and NEBF in Fig. 1b) where the 1980 Irpinia earthquake enucleated. Several works have suggested the presence of fluids in this crustal volume^2,15^, and in deeper volumes of other seismogenic zones of the southern Apennines chain^4,31^. These fluids are confined within the two boundary faults by the tectonic mélange, which can be considered a low-permeability barrier at the top of the Apulian platform. The physical rock up-scaling performed by Amoroso et al.^16^ showed that the deeper volume is highly fractured and that the pore fluid could be a combination of brine and CO_2_. Recently, Poli et al.^32^, by analysing the coda wave velocity variation in the region of the L’Aquila (Central Apennines), proposed the existence of an intensely cracked volume deep in the crust that is saturated in overpressured fluids. The hypothesis of overpressured fluids at depths, invoked in other works as primary mechanism of interaction between fluid dynamic and earthquake triggering^3,4,6,9^, is confirmed by our results. Indeed, the strongest changes in the Vp-to-Vs ratio in the deeper volume can be explained by considering the high sensitivity of rock elastic properties under overpressured conditions. Several experimental observations^33–36^ have indicated that Poisson's ratio, which is proportional to the Vp-to-Vs ratio, significantly decreases (increases) at very high effective pressure in gas (water)-saturated samples with tiny pressure changes.

We suggest that in the two volumes in the IFS, the observed seismic velocity changes are caused by different mechanisms: the first is related to meteoric water recharge processes in the shallower volume, and the other is related to pore pressure pulsations in the deeper volume.

To explain these mechanisms we schematically represented the medium behaviour in the two limited conditions of the discharge curve (Fig. 4b,c).

In the shallower volume, under conditions of limited recharge and low hydraulic head (epochs one and three, as indicated by grey arrows in Figs. 3b, 4b), we expect a decrease in meteoric water saturation in the aquifer rock matrix^37^. As observed in laboratory measurements^38^, Vs increases with decreasing water saturation, resulting in a decrease in the Vp-to-Vs ratio. From displacement measurements (Fig. 3c,d), the occurrence of negative displacement indicates that the shallower layer contracts horizontally (negative dilatation) in response to a decrease in hydraulic head. Alternatively, the increase in Vs could be explained by the closure of cracks and flaws, which elastically stiffen the rock matrix under horizontal contraction.

Assuming that the deeper volume is elastically coupled with the layer above, it will be solicited by equivalent negative horizontal dilatation^18^. This compression mechanism is likely to cause an increase in pore pressure in the highly fractured and fluid-filled volume. The consequent decrease in the Vp-to-Vs ratio indicates that the porous and fractured rock volume is dominantly saturated in gases (likely CO_2_ as inferred by gas emission analysis^4,7,9^).

Conversely, in wet conditions (Fig. 4c), the water saturation in the shallower volume increases, leading to a Vp-to-Vs ratio increase. Elastic coupling between the shallow and deeper volumes drives the last one into horizontal expansion that causes a decrease in pore pressure and a consequent increase in the Vp-to-Vs ratio.

In addition to the elastic coupling between the two layers, the velocity changes in the deeper volume could also be due to the diffusion of volumetric pore pressure perturbation induced by hydraulic head changes. The considered epoch time intervals do not enable us to track the pore pressure diffusion in time or to discriminate between the two mechanisms at depth.

The retrieved correlation between the velocity changes and the hydraulic head was found in other studies along the Apennines^32,39^ and worldwide^40,41^. However, in these cases, the velocity variations were measured through the coda wave time lapse or the ambient seismic noise cross-correlation allowing a higher temporal sampling rate (from hours to days). However, these measurements could not constrain the velocity anomaly in terms of its absolute amplitude and location, especially in the deeper region. Here, travel-time tomography, despite the low temporal sampling rate, allows us to evaluate with a greater accuracy the magnitude of Vp-to-Vs variations between epochs and to constrain the location of the fractured and overpressured volumes at depth.

The described hydrological forcing, stress perturbations and velocity changes represent important components of the IFS dynamic that may play a key role in triggering the current micro-seismicity. Although D’Agostino et al.^18^ found a strong, in-phase correlation between IFS seismicity during 2008–2018 and groundwater recharge in the entire IFS volume, we do not have enough data to explore this correlation since the seismicity rate is too low in the two volumes of interest.

However, the scale of seismic velocity variations in the IFS confirms the high sensitivity of medium elastic properties, also at depth, to the hydrological forcing of karst aquifers in the Southern Apennines. Our work suggests that tomographic measurements of the Vp-to-Vs changes with space and time enable us to track the pore pressure pulsations at depth, which occur within the 8–12 km deep, highly fractured and fluid-filled rock volume within the fault system and have caused the moderate to large earthquake occurrence during the past centuries.

## Materials and methods

For details about the method and inversion strategy, see the SI.

## Supplementary Information

Supplementary Information.
