# Author Correction: Decade-long monitoring of seismic velocity changes at the Irpinia fault system (southern Italy) reveals pore pressure pulsations

**DOI:** 10.1038/s41598-022-20912-2

**Published:** 2022-09-26

**Authors:** G. De Landro, O. Amoroso, G. Russo, N. D’Agostino, R. Esposito, A. Emolo, A. Zollo

**Affiliations:** 1 Department of Physics “E. Pancini”, University of Naples ‘Federico II’, Naples, Italy; 2 Department of Physics “E.R. Caianiello”, University of Salerno, Fisciano, SA Italy; 3 National Institute of Geophysics and Volcanology, Rome, Italy; 4 Formerly Department of Physics “E. Pancini”, University of Naples ‘Federico II’, Naples, Italy

Correction to: *Scientific Reports* 10.1038/s41598-022-05365-x, published online 24 January 2022

The Reference list in the original version of this Article was incomplete. The below Reference, which is now listed as Reference 11, was omitted.

DISS Working Group (2021). Database of Individual Seismogenic Sources (DISS), Version 3.3.0: A compilation of potential sources for earthquakes larger than M 5.5 in Italy and surrounding areas. Istituto Nazionale di Geofisica e Vulcanologia (INGV). https://doi.org/10.13127/diss3.3.0

As a Result, the caption of Figure 1,

“Tectonic setting of the Irpinia Fault System. (**a**) Geological sketch map. The white squares indicate the locations of the main historical and instrumental earthquakes; for the most recent earthquakes, the focal mechanisms are reported. The black boxes are the Irpinia Faults as reported by the Database of Individual Seismogenic Sources^34^. The green contoured areas represent the Apennine carbonate rocks. The contoured black area represents the aftershock area of the 1980 Irpinia earthquake. The dashed line is the SW–NE section shown in panel (**b**). The circles indicate the events used in this study that are coloured according to their depth. The map was generated with the Generic Mapping Tool Software (GMT ver.4; https://www.soest.hawaii.edu/gmt/). (**b**) Geological cross-section along the profile reported in the map in (**a**); *SWBF* SW boundary fault, *CF* central fault, *NEBF* NE boundary fault.”

now reads:

“Tectonic setting of the Irpinia Fault System. (**a**) Geological sketch map. The white squares indicate the locations of the main historical and instrumental earthquakes; for the most recent earthquakes, the focal mechanisms are reported. The black boxes are the Irpinia Faults as reported by the Database of Individual Seismogenic Sources^11^. The green contoured areas represent the Apennine carbonate rocks. The contoured black area represents the aftershock area of the 1980 Irpinia earthquake. The dashed line is the SW–NE section shown in panel (**b**). The circles indicate the events used in this study that are coloured according to their depth. The map was generated with the Generic Mapping Tool Software (GMT ver.4; https://www.soest.hawaii.edu/gmt/). (**b**) Geological cross-section along the profile reported in the map in (**a**); *SWBF* SW boundary fault, *CF* central fault, *NEBF* NE boundary fault.”

As a result of the changes, the References have been renumbered.

The original Article has been corrected.

