# Supplementary material for: Decade-long monitoring of seismic velocity changes at the Irpinia fault system (southern Italy) reveals pore pressure pulsations
Source: Sci Rep. 2022 Jan 24;12:1247. doi: 10.1038/s41598-022-05365-x (PMC8786847; doi:10.1038/s41598-022-05365-x)
Supplement: Supplementary file 1 — Supplementary Information. [file 41598_2022_5365_MOESM1_ESM.docx]

**Supplementary information of**

**Decade-long monitoring of seismic velocity changes at the Irpinia Fault System (southern Italy) reveals pore pressure pulsations**

**G. De Landro^1*^, O. Amoroso^2^, G. Russo^1^, N. D’Agostino^3^, R. Esposito^4^, A. Emolo^1^ and A. Zollo^1^**

^1^ Department of Physics “E. Pancini”, University of Naples ‘Federico II’, Italy.

^2^ Department of Physics “E.R. Caianiello”, University of Salerno, Fisciano (SA), Italy.

^3^ National Institute of Geophysics and Volcanology, Rome, Italy

^4^ Formerly department of Physics “E. Pancini”, University of Naples ‘Federico II’, Italy.

Corresponding author: Grazia De Landro (grazia.delandro@unina.it)

**Introduction**

The purpose of this supplementary materials, which are referenced in the manuscript as supporting information (SI), is to provide detailed information on the data processing, the tomographic inversion method, the setting of inversion parameters and the assessment of tomographic image resolution. The SI is organized in two sections, the first is dedicated to the 3D tomographic inversion considering the entire catalogue, the second contains details about the 4D tomographic inversions.

**3D Tomographic inversion strategy**

We used a linearized, tomographic approach in which the P and S arrival times are simultaneously inverted for the earthquakes location and velocity parameters [1]. As data-set, we used the ISNet (Irpinia Seismic Network) catalog from August 2005 to October 2016, consisting of 1948 low magnitude events (0.5<ML<3.2). For these earthquakes we used the manual picks revised by the maintenance team of ISNet, integrated with the picks available from the stations of Istituto Nazionale di Geofisica e Vulcanologia (INGV) network, located in the Irpinia Fault System (IFS) area. At each pick is assigned a weight based on the time uncertainty: the weight 0 corresponds to a time uncertainty lower than 0.05 s, the weight 1 to a time uncertainty between 0.05 s and 0.10 s, the weight 2 to a time uncertainty between 0.10 s and 0.20 s and weight 4 to a time uncertainty higher than 0.5 s.

We localized the events using the NLLoc [2] and the 1D velocity model retrieved for the IFS by Matrullo et al. (2013). Then, we selected the events with at least 4 P-wave and 2 S-wave picks, and azimuthal coverage of station (i.e. GAP) higher than 200°, and a localization RMS lower than 0.5 s. We ended with 1425 events providing 12598 P picks and 5834 S picks. The 65% of P-wave arrivals have weights between 0 and 1, on the other hand the 79% of S-wave arrivals have weights between 1 and 2 (see Fig. S1).

The starting velocity model for the inversion is the 1D model of Matrullo et al. [3], in which events were localized. These localizations are also used as starting points of the inversion. The inversion strategy was based on a multiscale approach [4]. The basic assumption is that large wavelength anomalies in the velocity structure have a dominant amplitude relative to the smaller ones, which we believe is reasonable at all scales of investigation within Earth. The initial inversion runs are performed using a low-resolution, high-wavelength parameterization of the medium, which is progressively refined by increasing the density of grid points at successive runs. The investigated rock volume of about 100x100x30 km^3^ was initially sampled with a 12x12x4 km^3^ regular grid. A first inversion was performed, then we reduce the grid steps and performed a new inversion starting from the velocity model obtained for the coarser grid (6x6x2 km^3^).

The inversion approach is based on an iterative scheme. First arrival travel times are computed through a finite difference solution of the eikonal equation [5] in a fine grid of 0.5 × 0.5 × 0.5 km^3^. Travel times for each event–receiver pair are recalculated by numerical integration of the slowness on the inversion grid field along the rays traced in the finite difference travel-time field. The parameters are inverted using the least squares root (LSQR) method of Paige and Sanders [6] based on a damped inversion. For the calibration of the damping factor we followed an empirical approach [7] based on L-curve construction: using real datasets of P and S arrival times, we performed several inversions for different values of damping. The data and solution variances are computed after one iteration for the indicated damping values. The chosen damping value, i.e. 0.6 for the inversion of the whole dataset (see Fig. S2), is the one for which a small variance in the data corresponds to a small variance of the model, for all four classes of parameters, simultaneously. The misfit function, defined as the sum of the squared time delay (RMS), is a posteriori analysed and we chose 15 iterations to define the convergence (Figure S3). The RMS reduction with the finest parameterization (red dots in Fig. S3) confirms ‘a posteriori’ that the adopted multi-scale strategy was the more efficient to explore the multidimensional model parameter space and to catch the minimum norm model solution.

The comparisons between histograms of travel time residuals in the initial (coarse) and the final (fine) velocity models is another evidence for it (fig. S4). The finest parameterized velocity model produces a peaked histogram of residuals centered at zero (e.g. a smaller data dispersion

around the a zero-mean value).

In Figure S5 we show the 3D P- and S-wave models and Vp-to-Vs model for the whole period obtained by using the two recursive parameterizations, cut in horizontal slices at different depths. The main large-scale features of the velocity models are retrieved in the first step of multi-scale inversion, with the coarser parameterization. The P- and S-wave coarser models (left panels of left and central columns in Fig. 6, respectively) reveal the strong lateral variation of velocity values, from 6 km of depth, that is then refined in the models obtained with the finer parameterization, from 4 km of depth. The Vp-to-Vs ratio coarser model (left panel in right column in Fig. S6) shows the high anomaly in the central portion of IFS, that is than well imaged in the finer parameterization.

We assessed the resolution of each output model by computing the full resolution matrix with the relation 11 of Rawlinson and Spakman [8]. Methods that quantify solution reliability explicitly, like calculation of full resolution matrix, should be preferred to sensitivity analysis in resolution assessment of a tomographic image [8]. The full resolution matrix is represented in terms of its resolution diagonal elements (RDE) and the spread function Sj [9] related to off-diagonal elements, i.e. a measure of the correlation between parameters. Complementary to the resolution matrix, we computed for each node the Derivative Weighted Sum (DWS; [10]) which measures the ray density in the neighbourhood of every node. All these quantities were then mapped together to determine the best resolved volumes and the corresponding RDE, Sj and DWS values. In this way, we are able to include in the resolution analysis of the single node its intrinsic resolution, the possible spreading due to the neighbouring nodes and the ray density around the node itself. In figure 2 of the main text and in figure S5 of SI, the black contour delimitates the resolved area, i.e. the area for which the tree resolution parameters (RDE, Sj and DWS) are higher than a threshold value. The threshold values of Sj (0 for P- and S-wave) and DWS (3000 for P-wave and 2000 for S-wave) are chosen in order to obtain similar contours to the 0.9 RDE one (Fig. S6). For the Vp/Vs ratio model we used the Vs well resolved contours. The resolution matrix and the spread function allow to state that the final model is well resolved down to 14 km of depth.

**4D Tomographic inversion strategy**

In order to obtain the 4D images, we divided the entire period into time windows (which we will refer to as “epochs''). We do not expect to have exactly the same extension of resolved volume in each epoch. The crucial aspect in our analysis is the comparison between velocity features that are inside the resolved area for all the epochs. So, to properly define the epochs duration, we required to have a good resolution (comparable with the one of the whole model) in the IFS volume of interest in each epoch. To fulfill this requirement we used the DWS values. As it is simple density ray measure and since the parameterization does not change between the epochs, DWS can meaningfully be compared between epochs.

For each epoch, we incremented recursively the event number and selected the one allowing to reach a good resolution in the volume of interest, and, at the same time, to maximize the temporal sampling. We evaluated the DWS matrixes and used the threshold values of the whole model to contour the resolved areas and check the epoch duration suitability. Moreover, for each epoch we evaluated the RDE and Sj and found that binding the DWS to the same values of the whole model (3000 for P-wave and 2000 for S-wave), the correspondent threshold values of RDE and Sj are 0.9 and 0, respectively for P- and S-wave.. In Figs. S7 and S8 we show the DWS for the obtained epochs and the RDE and Sj contours, at different depths and for P- and S-wave models, respectively. This procedure is feasible because the IFS back-ground seismicity insists on the same structure over time (Fig. S9), but, since it is non time-uniform, the epochs span different time periods (Table 1 in main text). In Fig. S10 we show the distributions of number and weights on P- and S-wave arrival times in each epoch.

In each epoch, we performed the tomographic inversion with the multi-scale approach. As initial model, we adopted the 1D velocity model obtained by averaging the 3D whole model, obtained with the coarser parameterization (12x12x4 km^3^), at each depth. We performed the optimization of inversion parameters for the first epoch and, then, we used the same setting in each epoch. This choice is feasible since the inversion frame (station/source layout, number of sources,…) is comparable in each epoch and the 4D tomographic analysis requires that the inversions in each epoch are made under the same settings to introduce no artifact velocity changes [11]. The chosen damping value, i.e. 0.9 (see Fig. S11), is the one for which a small variance in the data corresponds to a small variance of the model, for all four classes of parameters, simultaneously. The misfit function, defined as the sum of the squared time delay (RMS), is a posteriori analysed for each epoch and we assume the convergence at 15 iterations (Fig. S12). The comparisons between histograms of travel time residuals in the initial (coarse) and the final (fine) velocity models for each epoch confirms the convergence of iteration process (Fig. S13-S14). The 4D final velocity models produce a peaked histogram of residuals centered at zero (e.g. a smaller data dispersion around the a zero-mean value).

The comparison analysis is focused on two volumes of interest located in central region of IFS and characterized by Vp-to-Vs changes: one between 1 and 5 km of depth, named as “shallower”; and the other between 8 and 12 km of depth named as “deeper” (black boxes in Fig. 2c). To construct the trend of Vp-to-Vs ratio over the epochs, we calculated the cumulative distribution of Vp-to-Vs ratios values in the two volumes of interest, from which we, thus, extrapolated the values at 20%, 50% and 80% of the cumulative distribution of velocity (Figs. S15 and S16 for shallower and deeper volume, respectively). For each epoch, the values at 20% and 80% of the cumulative distribution are used to draw the confidence interval in Fig. 3a, on the other hand, the 50% is used as a main value (red and blue dot in Fig. 3a) of the Vp-to-Vs ratio trend over epochs.

In order to prove that our choice of epochs allows us to analyze the velocity temporal changes in equally resolved volumes and that there is not lack of resolution in 4D images, we carried out a sensitivity test. Indeed, if the resolution matrix can be computed, sensitivity analysis must be seen as a complementary means to assess how lack of resolution affects the retrieved model (Rawlinson and Spackman, 2016). We constructed a Vp/Vs synthetic model by adding two anomalies in the upper and lower volume, that mimic the Vp/Vs 3D whole model (Fig. S17a-b). Then, we calculated the synthetic data-set in each epoch by using the data-coverage inherited from the final 4D tomographic models. Finally, we inverted the data-set in each epoch, starting from the average 1D Vp/Vs model without the anomaly, and we found that the anomaly is well reconstructed in terms of location and intensity in each epoch (Fig. S17c-l). The variation between its intensity in the different epochs is lower than the variability that we observed in the Vp/Vs ratio temporal changes. We can conclude that, in the volumes that we analyzed, there is no lack of resolution in 4D images due to our choice of temporal duration.

**References**

1. Latorre, D. et al. A new seismic tomography of Aigion area (Gulf of Corinth, Greece) from the 1991 data set. *Geophys. J. Int*. **159**, 1013–1031, doi:10.1111/j.1365-246X.2004.02412.x (2004).
2. Lomax, A.J., Virieux, P., Volant & Berge, C. Probabilistic earthquake location in 3D and layered models: introduction of a Metropolis-Gibbs method and comparison with linear locations, in *Advances in Seismic Event Location*, pp. 101–134, eds Thurber, C.H. & Rabinowitz, N.,Kluwer (2000)
3. Matrullo, E., De Matteis, R., Satriano, C., Amoroso, O., &A. Zollo. An improved 1-D seismic velocity model for seismological studies in the Campania–Lucania region (Southern Italy). *Geophys. J. Int.* **195,** 460–473, doi: 10.1093/gji/ggt224 (2013).
4. Chiao, L.-Y., and B.-Y. Kuo , Multiscale seismic tomography, *Geophys. J. Int.*, **145**, 517– 527 (2001).
5. Podvin, P., & Lecomte, I.. Finite difference computation of traveltimes in very contrasted velocity models: a massively parallel approach and its associated tools. *Geophysical Journal International*, **105(1)**, 271-284 (1991).
6. Paige, C. C., and M. A., Saunders, LSQR, An algorithm for sparse linear equations and sparse least squares: ACM Trans. *Math. Software*, **8**, 43-71 (1982).
7. Eberhart-Phillips, D.. Three-dimensional velocity structure in northern California Coast Ranges from inversion of local earthquake arrival times. *Bulletin of the Seismological Society of America*, **76(4)**, 1025-1052 (1986).
8. Rawlinson, N. & Spakman, W. On the use of sensitivity tests in seismic tomography. *Geophysical Journal International***. 205(2),** 1221-1243 (2016).
9. Michelini, A. & McEvilly, T. V. Seismological studies at Parkfield. I. Simultaneous inversion for velocity structure and hypocenters using cubic B-splines parameterization. *Bulletin of the Seismological Society of America,* **81**, 524-552 (1991)
10. Toomey, D. R., & G. R. Foulger Tomographic inversion of local earthquake data from the Hengill Grensdalur central volcano complex, Iceland, *J. Geophys. Res*., **94**, 497–510 (1989).
11. Lumley, D. E.. Time-lapse seismic reservoir monitoring. *Geophysics*, **66(1)**, 50-53 (2001).


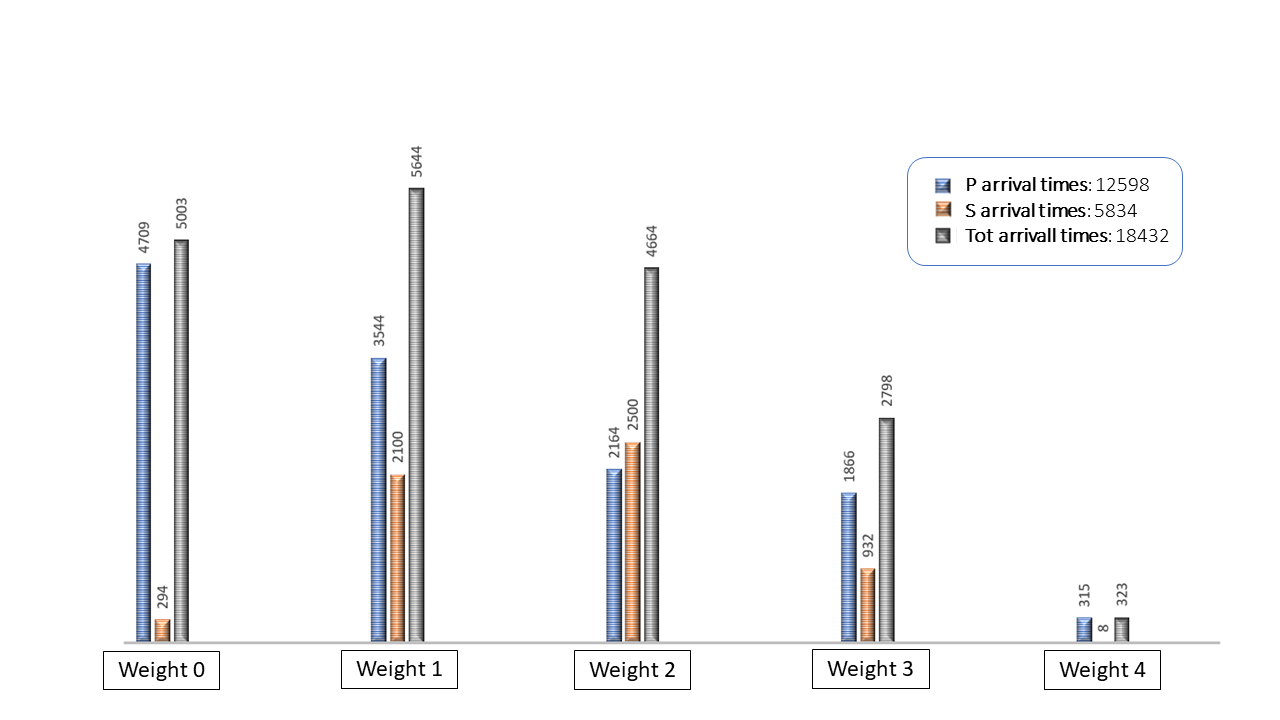


Figure S1. Arrival time number and weight distributions. Distribution of weight on arrival times of 1425 events recorded by the ISNet network, integrated with the INGV stations located in the IFS, from August 2005 to October 2016. The data-set consists of 12598 P-wave arrival times and 5834 S-wave arrival times. The blue columns represent the number of P-wave arrivals, the orange columns the S-wave arrival times and the grey columns the total number of arrival times for each weight. The 65% of P-wave arrivals have weights between 0 and 1, on the other hand the 79% of S-wave arrivals have weights between 1 and 2.


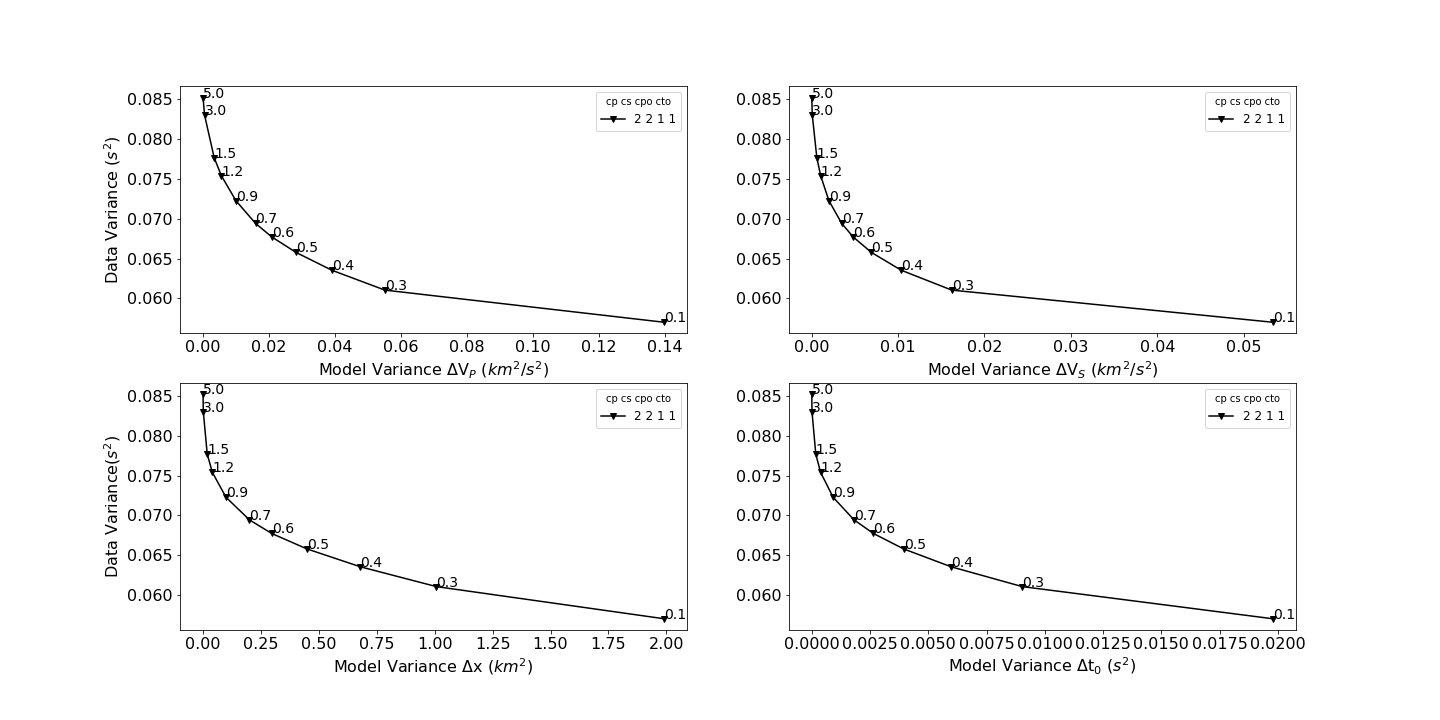


Figure S2. Trade off curves for selecting the optimal damping value for real data set. The panels show the L-curve obtained for each type of model parameter, i.e. the P-wave velocity (top left panel), the S-wave velocity (top right panel), the earthquake epicentral coordinates (bottom left panel) and the earthquake origin times (bottom right panel). The selected damping value is 0.6, suitable for all the model parameters.


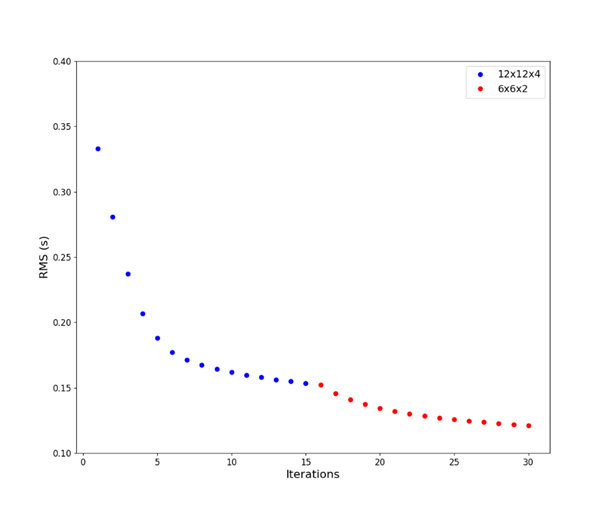


Figure S3. RMS vs iteration number for the two parameterization of multi-scale approach. Blue dots represent the RMS values obtained inverting P- and S-wave arrival times using the first parametrization of 12x12x4 km^3^, red dots correspond to the second parametrization of 6x6x2 km^3^.


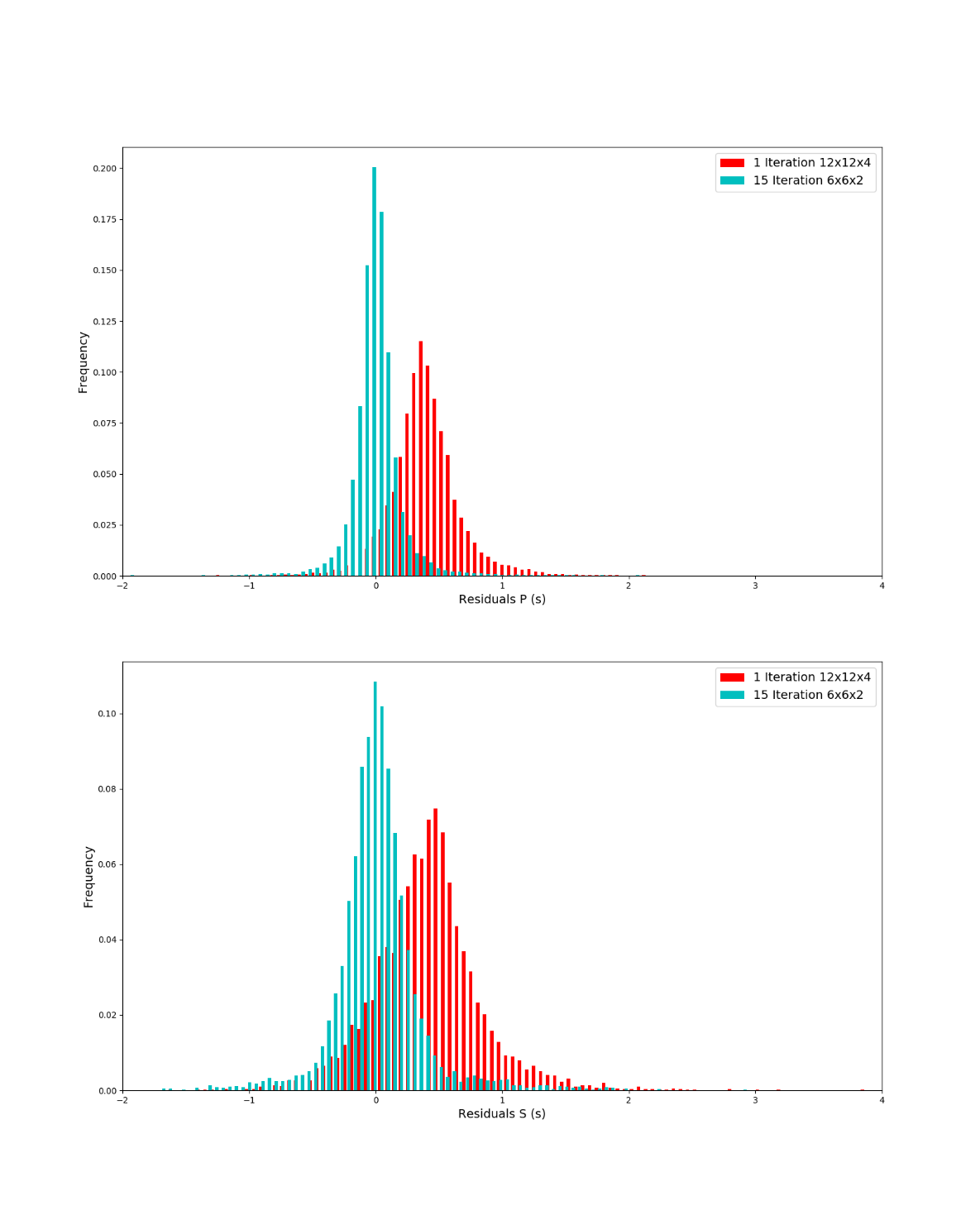


Figure S4. Initial (red) and final (blue) distributions of travel-time residuals of P-wave (top panel) and S-wave (bottom panel). The final residuals histogram shows a tight distribution centered on zero.


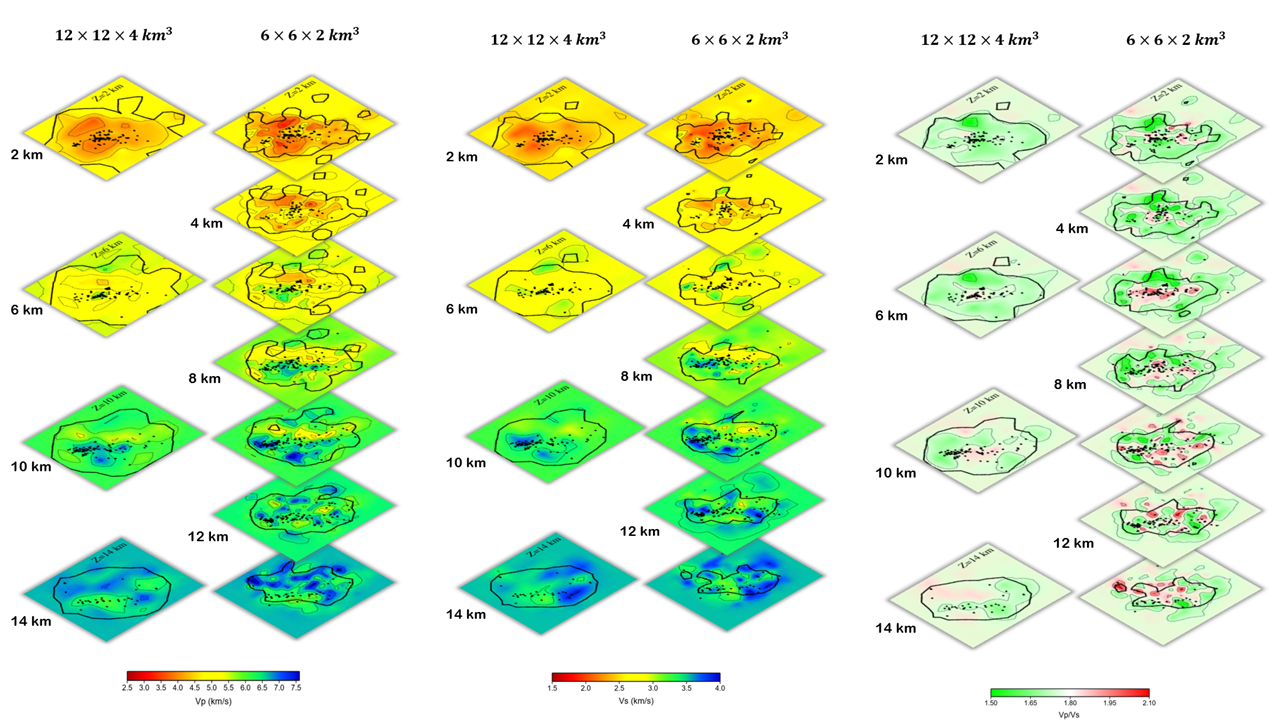


Figure S5. Map view at several depths of 3D velocity models obtained with the parametrizations 12x12x4km^3^ and 6x6x2 km^3^. In each slice, the black contour represents the resolved area by considering the resolution matrixes threshold values defined above.


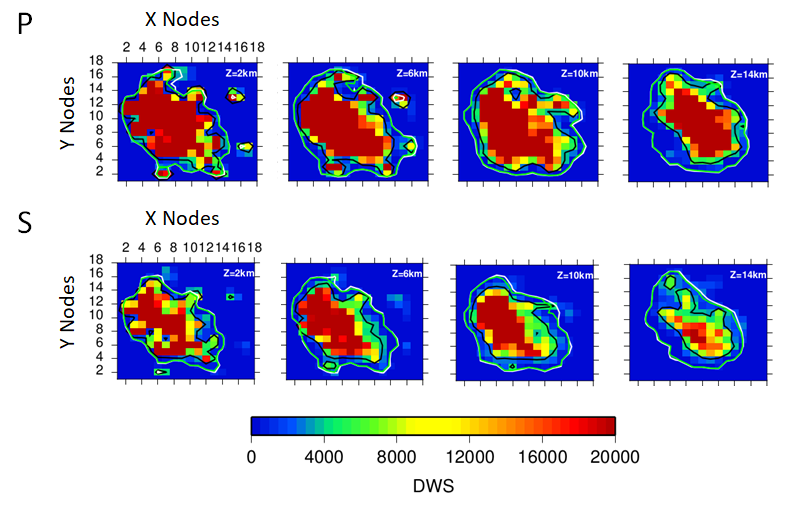


Figure S6. Resolution analysis for the 3D velocity model. The DWS matrix is represented at four different depths (i.e. 2, 6, 10 and 14 km) for the P-wave model (top panels) and S-wave model (bottom panels). In each panel the contours represents the resolved area based on threshold values of DWS (black), Sj (green) and RDE (white). These values are chosen in order to obtain a similar contour, binding the RDE to be higher than 0.9. The DWS thresholds are 3000 for P-wave model and 2000 for S-wave model.


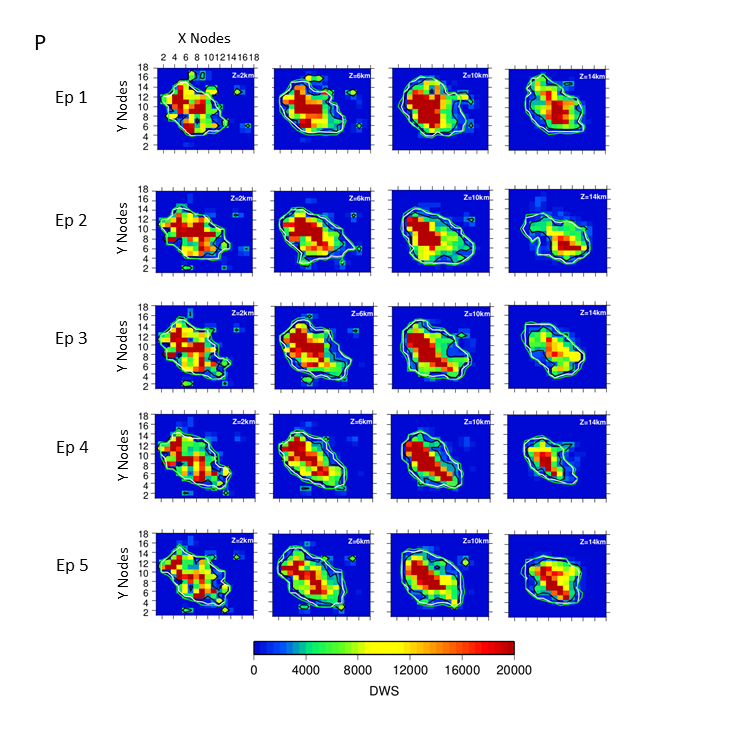


Figure S7. Resolution analysis for the 4D P-wave velocity models. For each epoch (indicated by the labels on the left), the DWS matrix is represented at four different depths (i.e. 2, 6, 10 and 14 km). In each panel the contours represents the resolved area based on threshold values of DWS (black), Sj (green, equal to 0) and RDE (white, equal to 0.9). These values are chosen in order to obtain a similar contour, binding DWS thresholds to 3000 for P-wave model, as for the whole 3D P-wave model.


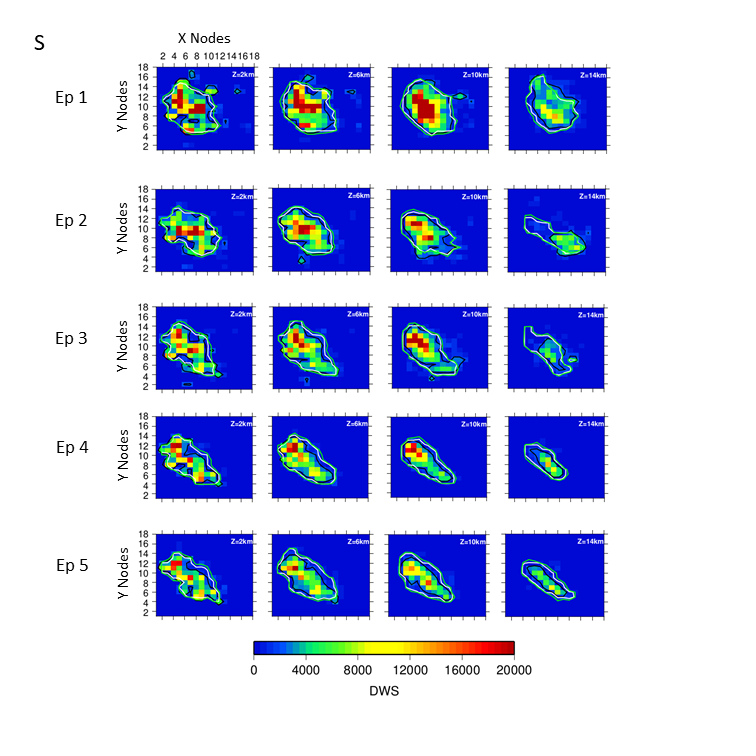


Figure S8. Resolution analysis for the 4D S-wave velocity models. For each epoch (indicated by the labels on the left), the DWS matrix is represented at four different depths (i.e. 2, 6, 10 and 14 km). In each panel the contours represents the resolved area based on threshold values of DWS (black), Sj (green, equal to 0) and RDE (white, equal to 0.9). These values are chosen in order to obtain a similar contour, binding the DWS thresholds to 2000 for S-wave model, as for the whole 3D S-wave model.


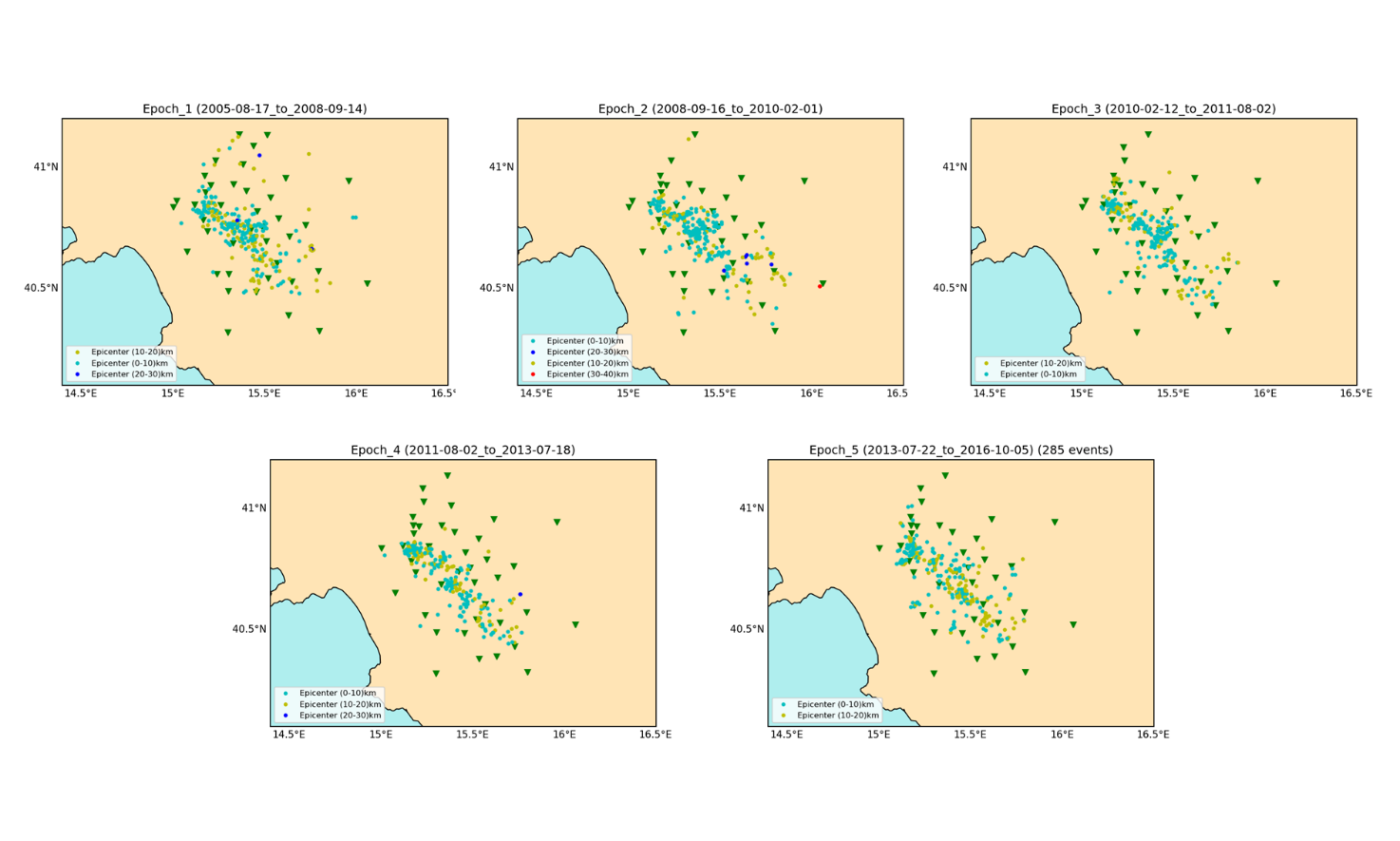


Figure S9. Event distribution in each epoch. Distribution of event epicenters (circles) in each epoch, as indicated in the panel title. The circle color represents the event depth.


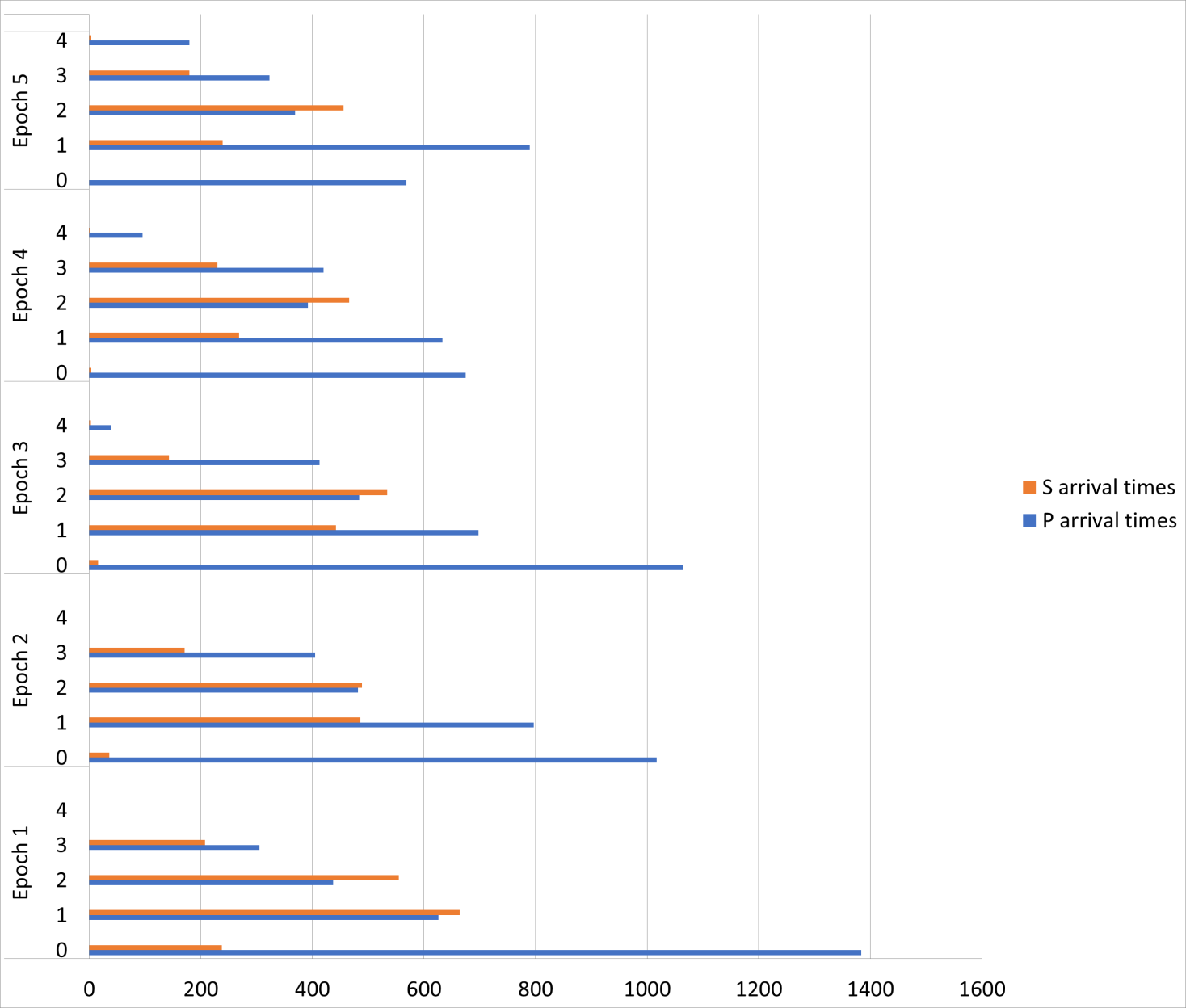


Figure S10. Arrival time number and weight distributions in each epoch. The data-set consists of 12598 P-wave arrival times and 5834 S-wave arrival times divided in 5 epochs. The blue columns represent the number of P-wave arrivals, the orange columns the S-wave arrival times for each weight, in each epoch.


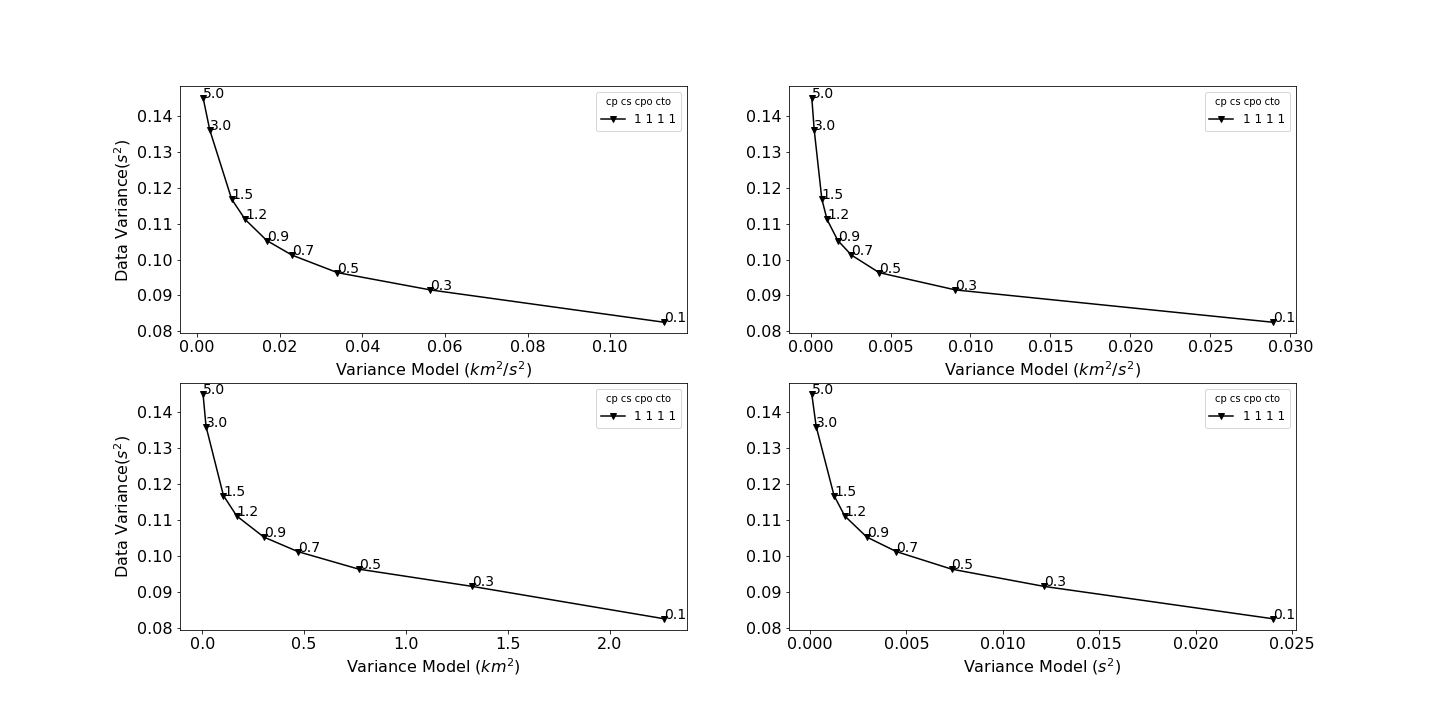


Figure S11. Trade off curves for selecting the optimal damping value for first epoch. The panels show the L-curve obtained for each type of model parameter, i.e. the P-wave velocity (top left panel), the S-wave velocity (top right panel), the earthquake epicentral coordinates (bottom left panel) and the earthquake origin times (bottom right panel). The selected damping value is 0.9, suitable for all the model parameters.


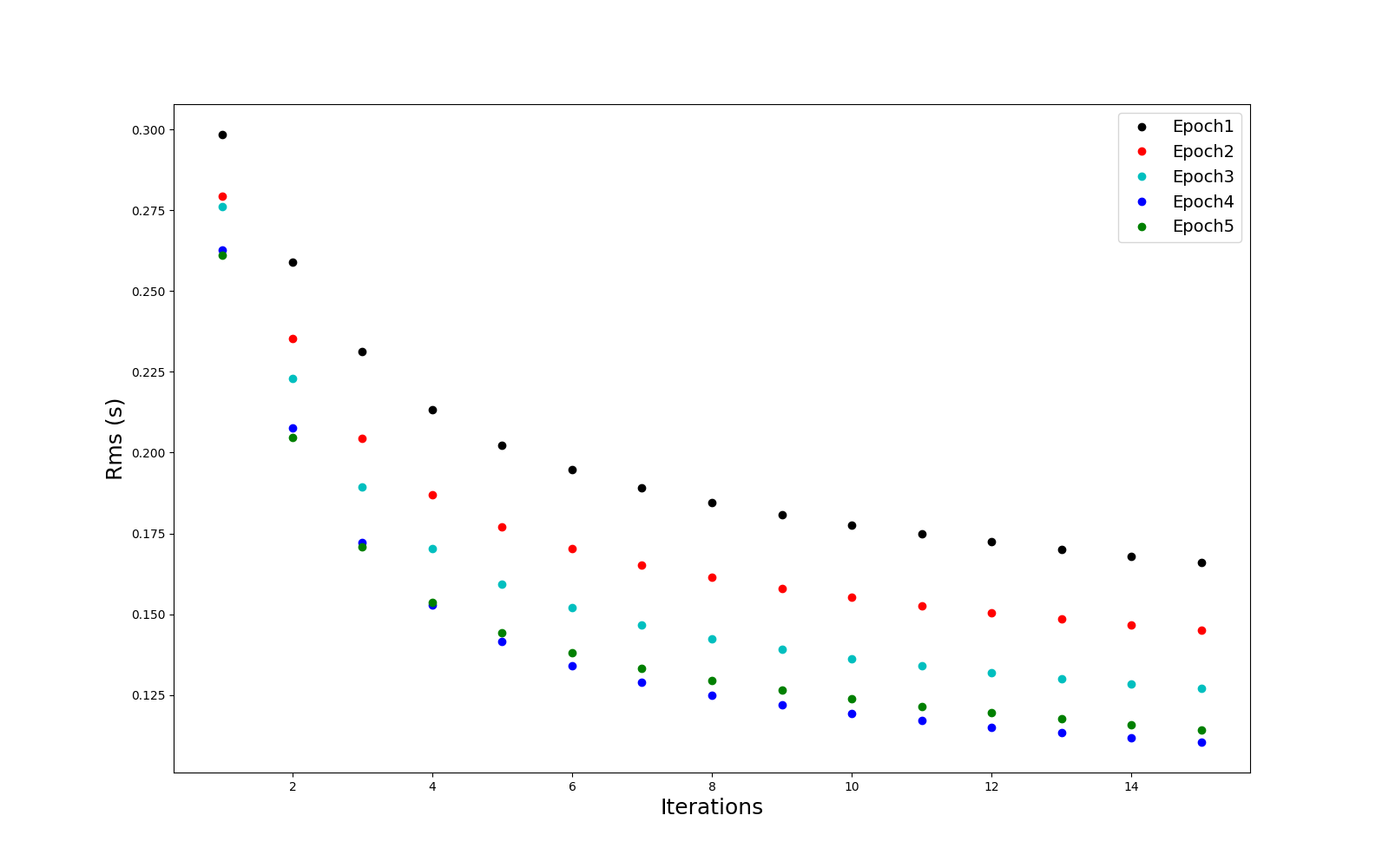


Figure S12. RMS vs iteration number for the epoch. The dot colors correspond to the epoch number: black to the first epoch, red to the second epoch, turquoise to the third epoch, blue to the fourth epoch and dark green to the last epoch. We chose 15 iteration to define the convergence.


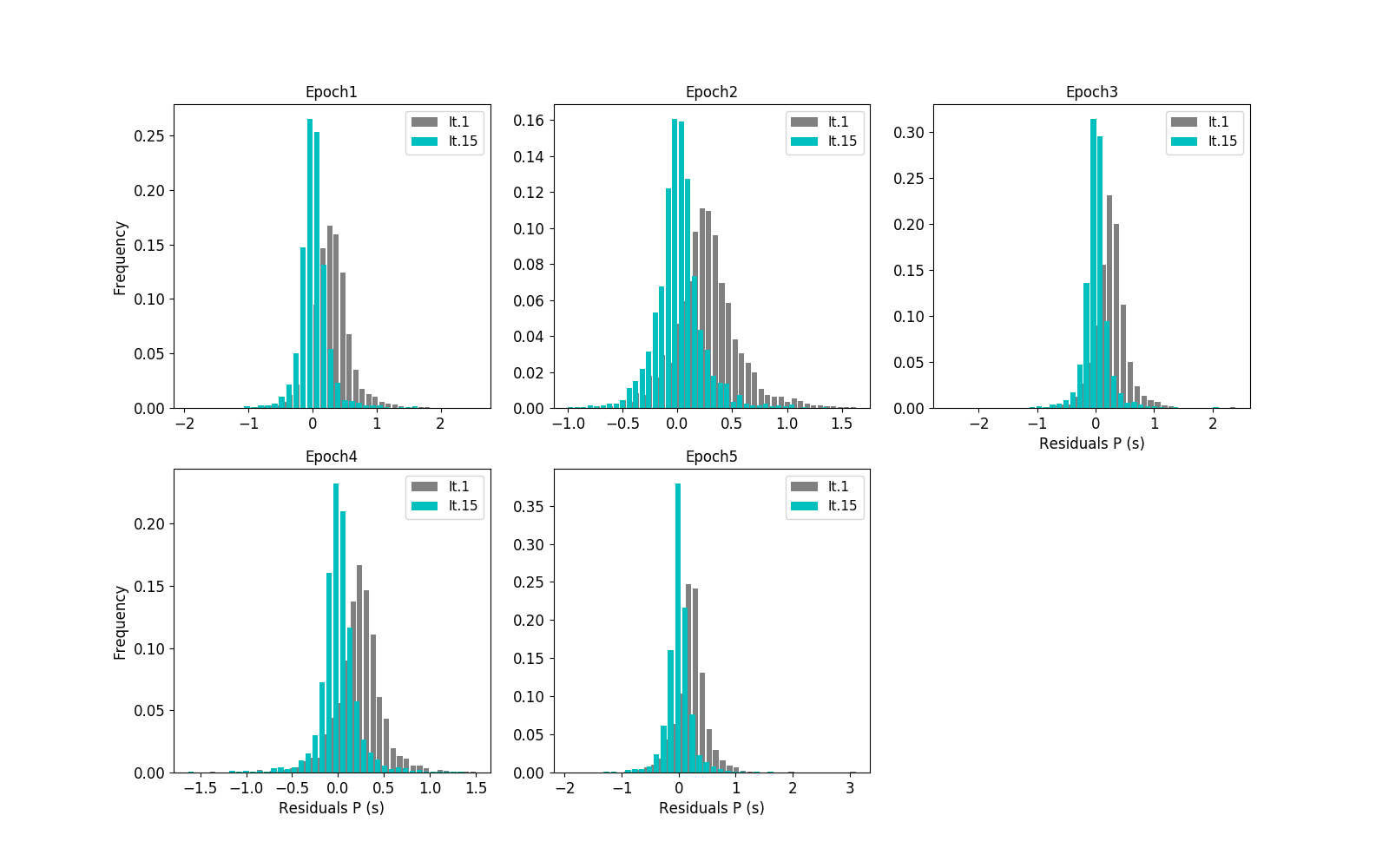


Figure S13. Initial (grey) and final (blue) distributions of P-wave travel-time residuals in each epoch, as indicated in the panel title. The final residuals histogram shows a tight distribution centered on zero in every epoch.


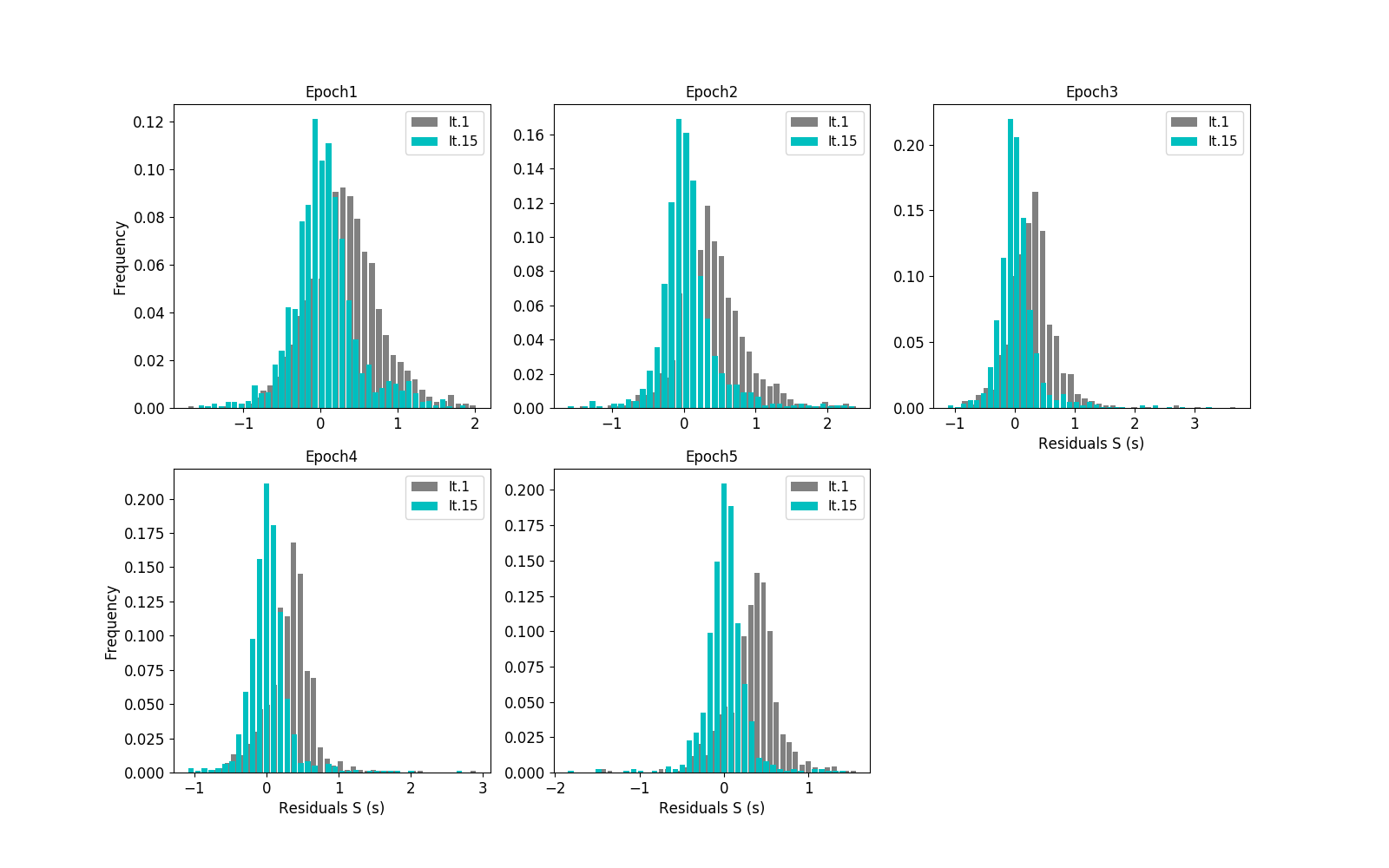


Figure S14. Initial (grey) and final (blue) distributions of S-wave travel-time residuals in each epoch, as indicated in the panel title. The final residuals histogram shows a tight distribution centered on zero in every epoch.


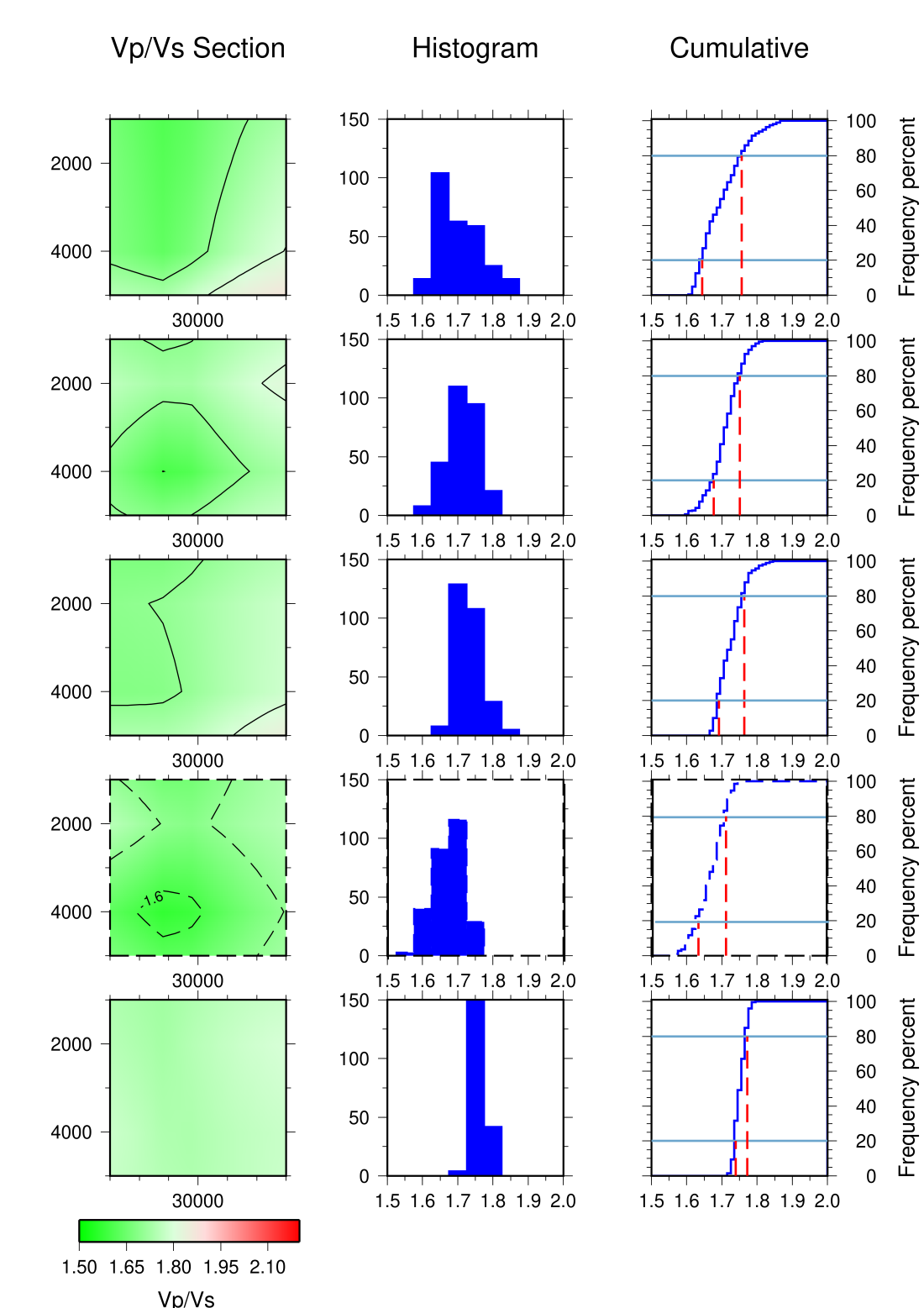


Figure S15. Extrapolation of Vp-to-Vs ratio trend in the shallower volume. Left panels represent the Vp-to-Vs tomographic sections (profile in Fig. 1a), at different epochs (from first at the top), focused in shallower volume. Central panels represent the histograms of Vp-to-Vs values in the shallower volume at different epochs (from first at the top). Right panels represent the cumulative distributions of velocity, from which we extract the values at 20% and 80% (red dashed segments) and the 50%.


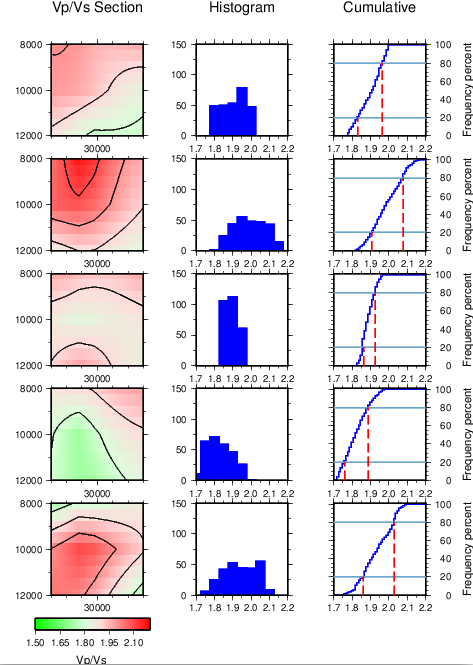


Figure S16. Extrapolation of Vp-to-Vs ratio trend in the deeper volume. Left panels represent the Vp-to-Vs tomographic sections (profile in Fig. 1a), at different epochs (from first at the top), focused in deeper volume. Central panels represent the histograms of Vp-to-Vs values in the shallower volume at different epochs (from first at the top). Right panels represent the cumulative distributions of velocity, from which we extract the values at 20% and 80% (red dashed segments) and the 50%.


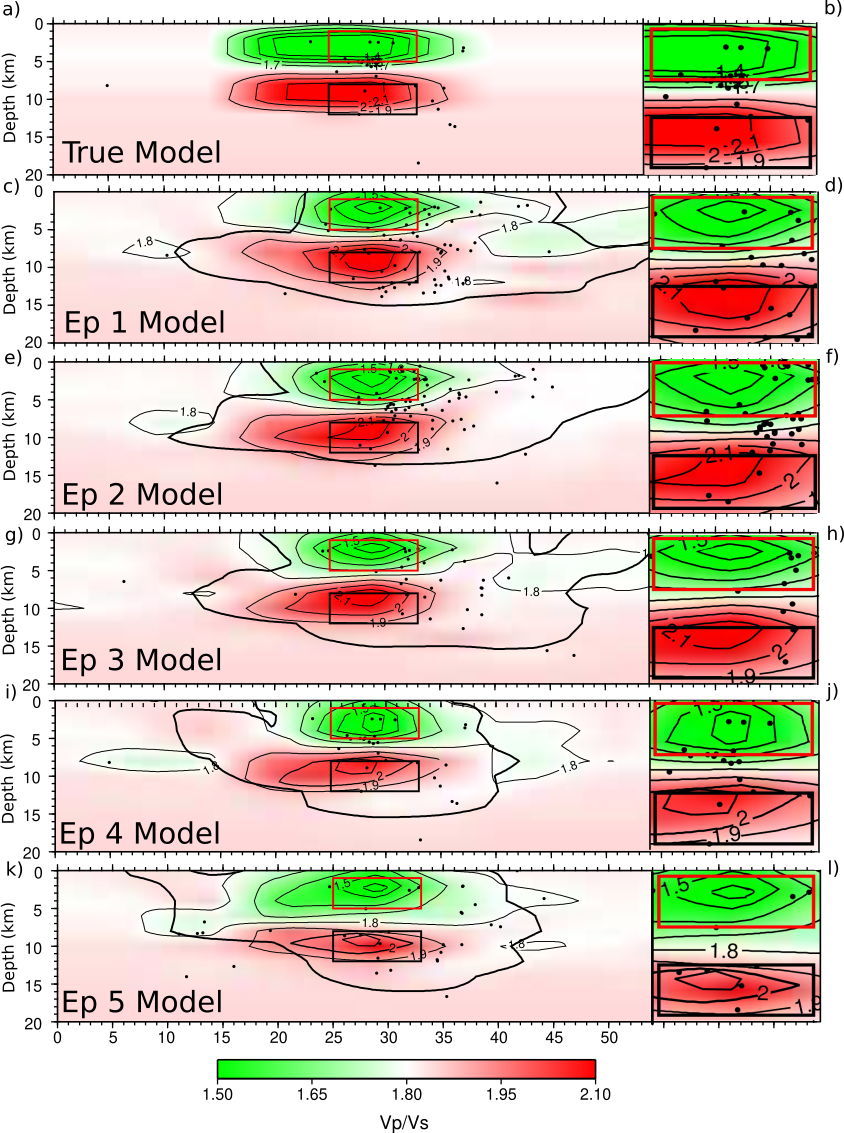


Figure S17. Results of sensitivity test. a) the true Vp/Vs anomaly along the profile in Fig. 1a of the main text. b) True Vp/Vs anomaly in the two investigated volumes (red, upper, and black, lower, square). Retrieved anomalies in the five epochs (c-e-g-i-k) and a focus of this anomalies in the two investigated volumes (d-f-h-j-l). In each section, the black contours enclose the resolved volume.
